# ReViT-RF: A hybrid ResNet-50–Vision Transformer with a Radiomic Feature Fusion framework for lung tumor classification and TNM-based stage prediction using CT images

**DOI:** 10.1371/journal.pone.0359211

**Published:** 2026-10-12

**Authors:** Shobharani DA, Ramesh Naik B

**Affiliations:** Department of Computer Science and Systems Engineering, GITAM School of Computer Science and Engineering, Bangalore, Karnataka, India; Hanyang University - ERICA Campus, KOREA, REPUBLIC OF

## Abstract

Lung cancer is one of the leading causes of cancer-related mortality worldwide, emphasizing the need for accurate, automated, and clinically interpretable diagnostic systems. Although existing deep learning (DL) models have achieved promising performance in classifying lung nodules as benign or malignant, most approaches are limited to binary classification and provide insufficient support for detailed tumor characterization and TNM staging. Furthermore, conventional convolutional neural networks (CNNs) are effective in extracting local morphological features but often fail to capture long-range spatial dependencies. In contrast, transformer-based models can learn global contextual information but may compromise fine-grained local feature representation. To address these limitations, this study proposes a hybrid ResNet-50–Vision Transformer with Radiomic Feature Fusion (ReViT-RF) framework for lung tumor classification and component-wise TNM stage prediction using thoracic computed tomography (CT) images from the publicly available Lung Image Database Consortium and Image Database Resource Initiative (LIDC-IDRI) dataset. The primary clinical objectives of the proposed framework are to: (i) classify pulmonary nodules as benign or malignant, and (ii) predict individual TNM components, including primary tumor extent (T), lymph node involvement (N), and metastatic status (M) for malignant cases. To improve tumor visibility and image quality, CT images are initially preprocessed using Contrast-Limited Adaptive Histogram Equalization (CLAHE) and intensity normalization. The proposed ReViT-RF architecture integrates the complementary advantages of ResNet-50 and Vision Transformer (ViT) for comprehensive feature learning. The CNN branch extracts local morphological, textural, and structural characteristics, whereas the transformer branch captures long-range spatial relationships and global contextual dependencies. In addition, handcrafted radiomic features, including shape, size, texture, and spatial descriptors, are fused with deep features to provide clinically meaningful information for accurate TNM component prediction. The dataset is divided into training, validation, and testing subsets using an 80:10:10 ratio, with data augmentation applied only to the training set to prevent information leakage. Experimental results demonstrate that the proposed framework achieves an accuracy of 97.62% for benign–malignant classification, along with high precision, recall, and F1-score values. Moreover, independent prediction of TNM components provides detailed stage-related information rather than relying on a single aggregated stage label, enabling more precise tumor assessment and clinical decision support. Furthermore, explainability analysis using Gradient-weighted Class Activation Mapping (Grad-CAM) identifies the critical tumor regions contributing to model predictions, improving interpretability and enhancing clinician confidence. Overall, by combining hybrid deep feature extraction, radiomic feature fusion, component-wise TNM stage prediction, and explainable AI analysis, the proposed ReViT-RF framework provides a reliable, comprehensive, and clinically interpretable solution for automated lung tumor analysis.

## Introduction

Lung cancer is a prevalent cancer type, affecting more than 2.5 million people in the world in 2022 [1]. It is the top death cause of all cancer related deaths, and it’s responsible for more than 18.7% of cancer deaths worldwide! According to estimates, there will be 125,070 lung cancer deaths in the US and 237,434 fatalities in the EU in 2024 [2]. The risk factors are air pollution, genetic susceptibility, diet, chronic inflammation and active or passive smoking. Other risk factors include exposure to ionising radiation, radon, heavy metals, asbestos and work-related exposure. Additionally, a higher risk of lung cancer and excessive alcohol consumption have been associated with certain medical conditions, such as HIV infection and pulmonary fibrosis [3].

Lung cancer is often diagnosed at a late stage, allowing few treatment options that could cure the disease, and thus has a high mortality rate. The percentage of cases identified at late stage (Stage III or IV) is around 85% at present [4]. The symptoms don’t usually manifest until the disease has developed well, and therapeutic interventions are therefore not as effective, making early and accurate detection systems very important. Hence, preventing and detecting lung cancer cases in time are crucial in lowering the incidence and mortality rates of the disease. The use of low-dose computed tomography (LDCT) of the chest has been found to be an effective screening method, especially for high-risk individuals who would otherwise not be identified without screening. The American Cancer Society recommends that people between ages 50 and 80 and who have a smoking history of at least 20 pack-years (cigarettes smoked per day multiplied by the number of years smoked) get screened for lung cancer annually [5]. The eligibility and exclusion criteria for lung cancer screening, however, differ between the different countries and regions of the member states of the European Union (EU).

Since lung cancer screening programs were implemented, there has been a notable improvement in the early diagnosis of lung cancer. Between 2013 and 2019, the incidence of lung cancer in the United States increased by 3.6% and 4.6% per year, respectively, at both localised and distant stages [6]. Similarly, after one year after the start of the lung cancer screening program, lung cancer mortality in South Korea was significantly lower (−2.69%; 95% CI: −4.24 to −1.13) and overall mortality was significantly lower (−3.21%; 95% CI: −4.84 to −1.58) [7]. The results show that screening programs enable early detection of cancer, which results in a favourable stage shift and hence an improvement in survival rates and cure rates.

The clinical staging procedure helps determine the best course of treatment for each patient in addition to tissue sampling and pathological analysis. To evaluate mediastinal lymph nodes, tissue samples are usually obtained using flexible videobronchoscopy (FVB), computed tomography (CT) guided percutaneous biopsy, or both with endobronchial ultrasound (EBUS) [8]. Video bronchoscopy is thought to be the preferred method for evaluating endobronchial tumors in the central airways while invasive percutaneous biopsy is the more effective procedure for peripheral lung lesions. The presence of the air-filled bronchus, which directly communicates or lies near to the lesion on computed tomography (CT) imaging (positive CT bronchus sign), is strongly related to the diagnostic success of guided bronchoscopy [9]. Bronchoscopy is crucial for the diagnosis of lung cancer, where tissue samples are taken for genetic profiling and histological confirmation, as well as for the study of immunological biomarkers [10]. If the metastatic disease is present, a second biopsy of the metastatic lesion may be necessary to characterize the lesion more. If they are not able to get a good tissue sample, other methods of diagnosis can be used, such as liquid biopsy techniques or cytological specimen analysis.

Clinical staging integrates the patient’s history, physical examination, metabolic imaging (CT), and minimally invasive surgical procedures (mediastinoscopy), as well as endoscopic procedures (bronchoscopy and endobronchial ultrasound and endosonography). These diagnostic tests are usually done in sequence, starting with the least invasive tests and moving to more invasive tests when clinically necessary [11].

### Problem statement

Although thoracic computed tomography (CT) has made recent progress, the diagnosis and staging of lung cancer is often difficult because its characteristics are quite variable and it is challenging to extract clinically relevant information to help with treatment planning. While the TNM staging process is widely carried out by manual review or by independent TNM staging systems, traditional deep learning techniques are essentially designed for binary classification problems. In addition, implementing CNN-based techniques into current architectures is a real dilemma, as they excel in capturing the long-range spatial relationships among CT images, but have not been well-suited to capturing fine-grained local features that are important in the detection of subtle tumor characteristics. Transformers, on the other hand, are not effective in capturing the long-range spatial relationships present in CT images. Thus, there is an urgent demand for an intelligent and interpretable framework which can simultaneously learn accurate tumor classification and reliable prediction of clinically relevant TNM components.

### Research objectives

The general aim of this research is to develop a hybrid deep learning system, named ReViT-RF, that combines lung tumor classification and estimation of the TNM stages of tumor components from computed tomography (CT) scans. The objectives are to:

- Designing a hybrid ResNet-50 and Vision Transformer network using local morphological feature extraction using CNNs and global contextual representation using Transformer networks for lung tumor analysis.
- Automated Lung Tumor Classification Framework based on Extracted Deep Features from CT Image.
- Identifying each individual component of the TNM stage for estimating tumor extent, lymph node involvement and distant metastasis, instead of estimating the overall cancer stage.
- Integrating radiomic features and deep image features to enrich the tumor morphological, textural, size and spatial characteristics in the prediction model.
- Using explainable artificial intelligence (XAI) via Grad-CAM approach to find the region of the image where the model is predicting.
- Evaluating the proposed framework to existing deep learning approaches quantitatively via performance metrics.

### Novelty and main contributions

- A Hybrid ReViT-RF architecture is proposed that combines the local morphological feature representation obtained by CNNs with the global context representation obtained from Transformers to comprehensively analyze lung CT images.
- Beyond classifying benign and malignant tumors, a multi-objective framework is designed to diagnose both types of tumors, and independently predict the T, N, M components for accurate tumor stage prediction.
- The Radiomic features and deep features are fused to get complementary tumor characteristics such as morphology, texture, size and spatial location, supporting TNM component prediction and classification.
- To enhance the transparency and interpretability of automated decisions, an Explainable AI approach using Grad-CAM is used to offer visual explanations of the image regions that are important for the model’s predictions.
- The proposed work is extensively applied to lung tumor classification and the prediction of TNM components, and compared with the state-of-the-art deep learning methods using proper evaluation metrics.

## Related works

With recent advances in artificial intelligence, numerous methods have been developed to analyze lung tumors automatically from computed tomography (CT) images. The current studies have mainly been aimed at the segmentation of lung tumors and the differentiation of benign and malignant tumors. Cancer staging and incorporation of radiomic and deep-learning features are now receiving more attention. Various CNN-based, Transformer-based, radiomic, and hybrid methods were investigated to further improve tumor characterization and prediction performance. This section provides a detailed account of these existing methods.

The automated deep learning-based segmentation method, called Incremental Multiple Resolution Residual Network (iMRRN), was developed by Dunn et al. [12] based on the Lung-PET-CT-Dx dataset from The Cancer Imaging Archive (TCIA), which is an open-access medical imaging repository. The data set consists of CT scans of 355 lung cancer patients. The iMRRN model achieved a success rate of 95.65% in the cancer lesion segmentation task in non-contrast computed tomography (CT) scans. Seven classifiers were subsequently used to classify the segmented lesions into different subtypes of lung cancer based on the radiomic features extracted from the segmented lesions. Oversampling methods were used to correct the class imbalance. The most important textural features for discriminating between lung cancer subtypes were the higher order features that were determined by the chi-square method.

Naseer et al. [13] suggested a computational intelligence-based method to automatically detect and classify lung cancer from CT scans. The framework consists of three stages: lung lobe segmentation, nodule identification, and benign–malignant classification. The first step involves segmenting the lung lobes from CT scans using a modified U-Net model. The resulting masks and labels will then be fed to an enhanced U-Net model for further processing and identification of potential pulmonary nodules. Lastly, a Support Vector Machine (SVM) classifier classifies the detected nodules as benign or malignant. Besides, the modified AlexNet architecture is added to further improve the overall classification performance.

Mohamed and Ezugwu [14] created a novel deep learning technique for lung cancer diagnosis by incorporating mRNA, miRNA, and DNA methylation biomarkers. Differential expression analysis and multi-step data pre-processing are the first steps in the proposed strategy for identifying genes with distinct expression patterns in different lung cancer stages. The tool DESeq2 was used to analyse RNA-seq data, whereas the package LIMMA was used to analyse DNA methylation and miRNA data. A combined dataset including 448 samples and 8,228 gene characteristics was produced by merging three omics datasets based on shared samples. Principal Component Analysis (PCA) was used to reduce feature dimensionality, while the Synthetic Minority Over-sampling Technique (SMOTE) was used to solve the issue of class imbalance. In the end, the PCA-SMOTE-CNN model was used to classify the merged dataset.

Wahengbam and Sriram [15] highlighted the significance of precise lung cancer identification and the efficacy of treatment planning. Lung cancer segmentation can be made more automated and accurate by computational techniques, which will help patients receive better care and an earlier diagnosis. Three deep learning classifiers CNNs, stacked autoencoders, and Recurrent Neural Networks (RNNs) were used by the researchers to categorise lung cancer from CT scan pictures. By assessing the sensitivity, specificity, and accuracy of these models, their performances were examined and contrasted.

Malligeswari and Kavya [16] developed a model called Res-U-Net that uses molecular imaging to categorise different forms of lung cancer. The method makes use of PET/CT scans of ^18F-FDG, which are widely utilised in oncology and have both anatomical and metabolic content. The radiogenomics dataset of the ^18F-FDG PET/CT images is used by the proposed Res-U-Net to classify small-cell lung cancer (SCLC), non-small-cell lung cancer (NSCLC), and normal patients. PET/CT scan pre-processing uses Gaussian smoothing to remove noise. The Support Vector Machine (SVM) classifier is used to further analyse the features gathered from the given model in order to obtain the final classification result, which shows the effectiveness of the provided technique.

Kashyap et al. [17] classified histological pictures of lungs into three classes: squamous cell carcinoma, adenocarcinoma, and normal tissue. They then applied three of the most recent deep neural network designs, ResNet-50 V2, EfficientNetB1, and VGG-19, to improve the accuracy and reliability of the histopathological diagnosis of LC. A convolutional neural network, also known as a pretrained model, has been used successfully in a variety of computer vision applications, including object detection, semantic segmentation, instance segmentation, and image classification. These models can also be used for picture synthesis, segmentation, and acquisition.

Kasinathan G, Jayakumar S In [18] a hybrid approach, Cloud-LTDSC (Cloud Lung Tumor Diagnosis and Staging), for diagnosis and staging of lung cancer in PET/CT images was proposed. The proposed model is mainly based on segmentation method using active contour to extract the lung tumor and classification method using an improved multi-layer convolutional network (M-CNN) to classify the various stages of lung cancer. The model was tested with 50 patients with a low dose (standard LIDC-IDRI data set) lung CT DICOM images. The experimental outcomes have shown the proposed method’s improved accuracy, recall, and precision compared to other methods.

Paramasivam et al. [19] used medical imaging to accurately identify disease-affected regions. They proposed A-CapsNet, an attention-based capsule network integrated with distributed dynamic routing, in which the attention layer continuously analyzes the feature vectors of digit capsules. The study was conducted using datasets from LIDC-IDRI, the Lung Nodule Analysis 2016 (LUNA16) challenge, and The Cancer Imaging Archive (TCIA). Initially, a Generative Adversarial Network (GAN) was employed to enhance image resolution. Subsequently, VGG-16 was used to extract high-level texture features, while the Gray-Level Co-occurrence Matrix (GLCM) was applied to extract low-level structural features.

Cheung et al. [20] created artificial intelligence algorithms to predict the overall stage of NSCLC using patient demographic information and radiomic features from initial CT images. Patients with advanced-stage disease can be prioritised for histological examination using the suggested strategy. There were two patient cohorts in the trial. Thirty percent of the samples in the NSCLC-Radiomics dataset from The Cancer Imaging Archive were used for internal testing, while the remaining seventy percent were used for training. For external validation, a different patient group from a nearby hospital was used. Following the delineation of the gross tumour volumes by clinical oncologists, 107 radiomic characteristics were retrieved and integrated with patient demographic data. To categorise patients into stages I, II, and III, a feed-forward neural network was created. Four traditional machine-learning classifiers were used to compare its performance. The accuracy of the suggested model was 88.84% during validation, 76.67% on the internal test set, and 74.52% on the external test set.

### Research gaps

- Existing studies using the LIDC-IDRI dataset have primarily focused on benign–malignant classification, with limited emphasis on clinically significant multi-class tumor staging.
- Convolutional Neural Networks (CNNs) effectively learn local morphological features, whereas Transformers capture global contextual dependencies. However, their integrated application for lung tumor characterization remains insufficiently explored.
- The combined utilization of radiomic descriptors and deep learning-based representations for comprehensive tumor characterization has received limited attention.
- Existing approaches provide inadequate visual interpretation of prediction-relevant tumor regions, limiting model transparency and clinical interpretability.
- Comprehensive benchmarking against state-of-the-art methods using consistent evaluation criteria remains insufficiently addressed.

## Proposed method

The proposed ReViT-RF architecture for automated lung tumor analysis in thoracic CT images as shown in Fig 1 is an integrated architecture. The architecture is composed of three modules, ResNet-50 for morphologic tumor characteristics extraction, Vision Transformer for contextual tumor extraction, and radiomic feature fusion for quantitative tumor extraction, thus allowing the extraction of complementary tumor characteristics. The overall workflow of ReViT-RF consists of five steps as shown in Fig 1: (i) CT image preprocessing, (ii) hybrid deep feature extraction, (iii) radiomic feature extraction and feature fusion, (iv) benign–malignant classification and TNM stage prediction, and (v) explainability analysis using Grad-CAM.

**Fig 1:**
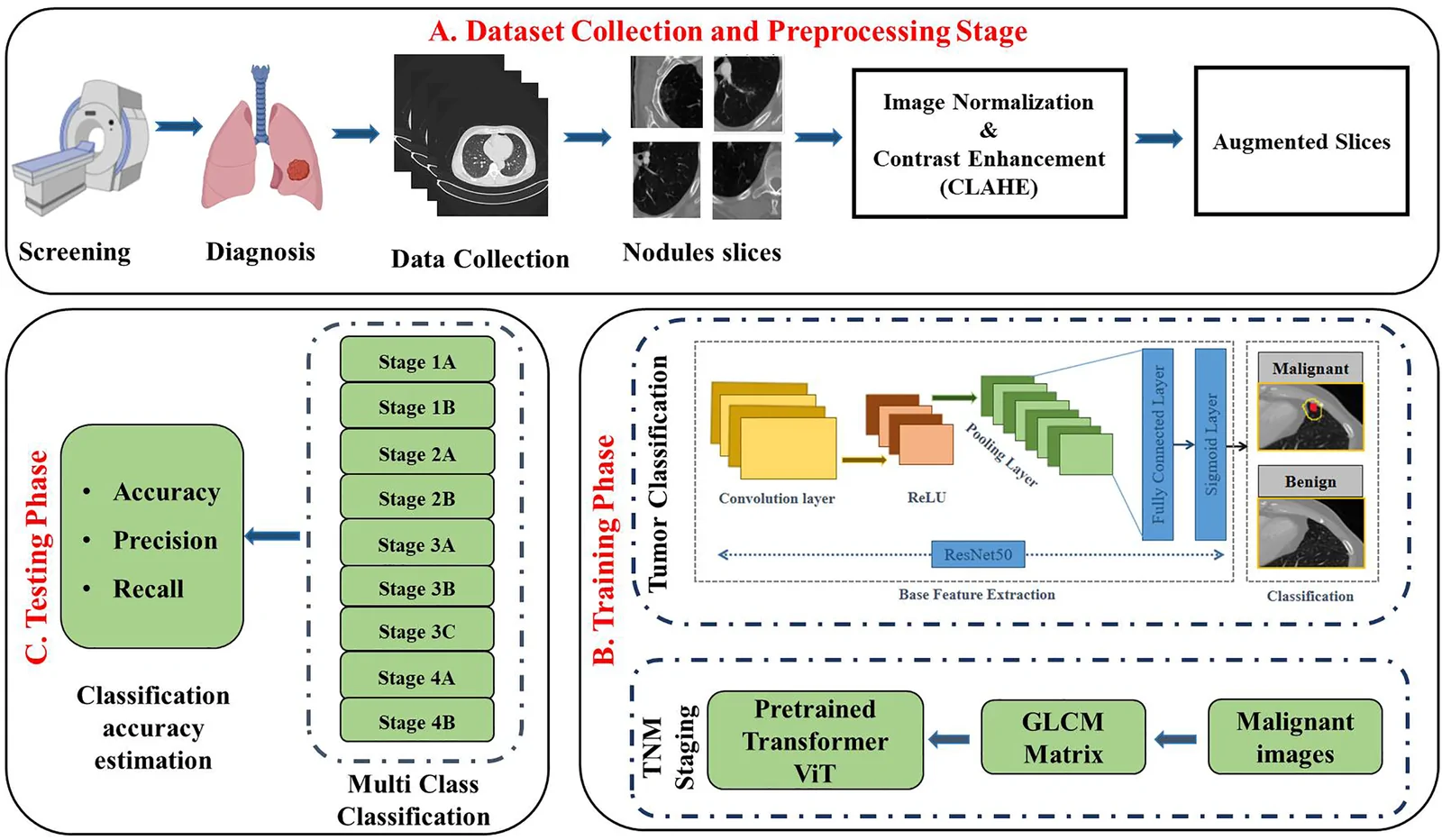
A Schematic diagram of proposed ReViT-RF model for lung tumor analysis.

At first, the input CT images are normalized according to the intensity and then enhanced by the use of CLAHE. The preprocessed CT images are then processed through two parallel deep learning branches. The ResNet-50 model learns discriminative local representations that correspond to tumor morphology, texture and structural characteristics in the first branch, while the second branch, built on the Vision Transformer, uses a self-attention mechanism to take into account the global context of various image regions. The full representation is then created by combining the deep features with manually created radiomic features. It is employed in a fused feature representation to categorise benign or malignant tumours and predict the cancer stage based on the T, N, and M components. To conclude, the Grad-CAM method provides visual explanations of the image regions that are important for the network’s predictions. The accuracy, precision, recall, F1 score, and detailed classification analysis are used to statistically evaluate the performance of the proposed framework. To sum up, ReViT-RF can be utilised as an integrated system for lung tumour analysis that integrates deep feature learning, radiomic feature fusion, interpretable visualisation, and stage prediction based on TNM components.

### Dataset and data-splitting

To test the proposed framework, the publicly available LIDC-IDRI dataset [21] was used, which consists of 1,018 MRI scans of the thorax from over 1,010 patients obtained from seven academic medical centers. About 244,000 CT slices and annotated pulmonary nodules were obtained from four experienced thoracic radiologists. Each nodule with the annotations has details about its location, contour, size, shape, texture, and likelihood score for malignancy (1 = very unlikely to be malignant; 5 = very suspicious of malignancy). XML annotation files were used by the radiologists to locate and retrieve the pulmonary nodules >3 mm in diameter.

The study involved complete radiological annotations, clear delineation of lesions and acceptable CT image quality with pulmonary nodules. Scans were excluded when they were missing annotations, if they had severe imaging artefacts, if there was still information missing about the nodules or if the contours of the lesions were not suitable for analysis. Nodules that had low malignancy ratings were considered as benign, while those with high malignancy ratings were considered as malignant. Samples with intermediate malignancy scores were removed from the binary classification task, which helped alleviate label uncertainty.

The dataset was first split into training, validation, and testing subsets at the patient level using an 80:10:10 ratio in order to prevent data leaking. The same patient’s CT slices and nodules were all allocated to a single subset. Therefore, no patient, CT image, or nodule appeared in more than one data partition. All preprocessing and data augmentation operations were performed only after dataset partitioning to maintain the independence and reliability of the validation and testing datasets.

Although LIDC-IDRI is widely used for pulmonary nodule classification, it does not provide complete pathological TNM staging labels. In particular, definitive annotations for lymph-node involvement and distant metastasis are unavailable. Therefore, the present study performs imaging-assisted prediction of TNM components using lesion dimensions, volumetric measurements, radiomic features, spatial information, and available radiological annotations. The T component is estimated based on nodule size and volume according to established tumour-size criteria, whereas the N and M components are considered imaging-derived proxy labels based on the available CT indicators. These predictions are intended exclusively for clinical decision-support analysis and should not be considered substitutes for pathological TNM staging, which requires biopsy confirmation, PET/CT evaluation, and comprehensive clinical assessment.

The dataset can be signified as:


$$\begin{array}{c} D={\left\{(X_{i},A_{i})\right\}}_{i=\text{1}}^{N} \end{array}$$ (1)

Where ${\text{X}}_{\text{i}}$ denotes the CT image of the ${\text{i}}^{\text{th}}$ patient, ${\text{A}}_{\text{i}}$ represents the corresponding expert annotation, and N denotes the total number of CT samples. The annotations provide valuable clinical information for lung nodule analysis and subsequent tumor classification.

### Data preprocessing

Each data partition was processed independently of the others for all the preprocessing operations. The preprocessing parameters, such as the intensity normalization statistics, were also calculated using only training subset. The mean and standard deviation of the training images were used to specifically normalize the training, validation, and testing images as follows:


$$\begin{array}{c} \hat{x}=\frac{x-\mu_{train}}{\sigma_{train}} \end{array}$$ (2)

Where $\mu_{\text{train}}$ and $\sigma_{\text{train}}$ denote the mean and standard deviation computed from the training data, respectively. The testing and validation subsets were then subjected to the same fixed parameters.

To reduce intensity variations caused by differences in CT scanners and acquisition protocols, z-score normalization is applied to each CT image:


$$\begin{array}{c} X_{i}^{norm}=\frac{X_{i}-\mu_{i}}{\sigma_{i}} \end{array}$$ (3)

Where ${\text{X}}_{\text{i}}^{\text{norm}}$ represents the normalized CT image, while $\mu_{\text{i}}$ and $\sigma_{\text{i}}$ represent the mean intensity and standard deviation of the ${\text{i}}^{\text{th}}$ CT scan, respectively. This normalization ensures a consistent intensity distribution, enabling effective model training.

After normalization, CLAHE is applied to enhance local contrast and improve the visualization of small tumor boundaries.


$$\begin{array}{c} X_{i}^{enh}=CLAHE(X_{i}^{norm}) \end{array}$$ (4)

Where ${\text{X}}_{\text{i}}^{\text{enh}}$ denotes the contrast-enhanced CT image. CLAHE improves the visibility of low-contrast nodules by enhancing local intensity variations while minimizing noise amplification.

To focus the model on the clinically relevant lung nodule region, segmentation is performed using either threshold-based or deep learning-based methods. The segmented Region of Interest (ROI) is defined as:


$$\begin{array}{c} X_{i}^{seg}=X_{i}^{enh}⊙M_{i} \end{array}$$ (5)

Where ${\text{M}}_{\text{i}}$ represents the binary lung nodule mask, and ⊙ denotes element-wise multiplication. The segmentation process removes irrelevant background information, enabling the network to focus on tumor-specific regions.

To make the segmented CT images compatible with the input requirements of the deep learning models, they are resized to a uniform spatial resolution of 224 × 224 pixels.


$$\begin{array}{c} X_{i}^{res}=R(X_{i}^{seg},\text{2}\text{2}\text{4}\times \text{2}\text{2}\text{4}) \end{array}$$ (6)

Where R(·) indicates the image resizing operation. This step ensures uniform input dimensions and improves computational efficiency during the training process.

Lastly, data augmentation (rotation, horizontal flip, translation, scale and intensity variation) was performed on training set only. There was no augmentation of the validation set or test set. This procedure guaranteed the validation samples and test samples were independent, and that they contained data which was not seen at the time of the model evaluation. Nodule cropping, resizing and intensity transformations were carried out independently for each data subset according to the patient-level assignments. Different image transformation techniques were applied to the CT samples to create augmented CT samples.


$$\begin{array}{c} X_{i,j}^{aug}=T_{j}\left(X_{i}^{res}\right),\;j=\text{1},\text{2},\ldots ,m \end{array}$$ (7)

Where ${\text{T}}_{\text{j}}(\cdot )$ represents the augmentation transformations, and m denotes the number of applied transformations. These augmentation strategies enhance the robustness and generalization capability of the ReViT-RF method, enabling it to detect lung nodules with varying sizes, orientations, and anatomical locations more effectively.

### TNM staging criteria

Table 1 provides the TNM staging criteria. The TNM approach, which takes into account the tumor’s size (T), involvement of the lymph nodes (N), and presence of metastases (M), is used to stage lung cancer. Tumours up to 3 cm in size that do not involve any lymph nodes (LN0) are classified as stages IA1–IA3. Tumours up to 4 cm in size that do not involve any lymph nodes are classified as stage IB. Stage IIA: The tumours have not migrated to the lymph nodes (LN0) and have a diameter of 4–5 cm. Stage IIB: The tumours are larger than 5 cm in diameter and have somewhat migrated to the lymph nodes (LN1). Larger tumours and/or progressed lymph node spread (mediastinal LN or contralateral LN2/LN3) are characteristics of stages IIIA–III C. Even with tiny initial tumours, stages IVA and IVB are the most advanced stages of the disease and are linked to distant metastases (DM1/DM2), nodules in the opposing lung, and malignant effusion in the heart or pleura.

**Table 1:** Stages of TNM.

Lung Tumor Stage	Primary Tumor (T/TU) – Tumor Size and Characteristics	Lymph Node Involvement (N/LN)	Distant Metastasis (M/DM)
Stage 0	TU0 – No evidence of primary tumor	LN0 – No lymph node involvement	DM0 – No distant metastasis
Stage IA1	T1a – Tumor size ≤ 1 cm	LN0 – No regional lymph node metastasis	M0 – No distant metastasis
Stage IA2	T1b – Tumor size >1 cm and ≤2 cm	LN0	M0
Stage IA3	T1c – Tumor size >2 cm and ≤3 cm	LN0	M0
Stage IB	T2a – Tumor size >3 cm and ≤4 cm	LN0	M0
Stage IIA	T2b – Tumor size >4 cm and ≤5 cm	LN0	M0
Stage IIB	T2/T3 – Tumor size ≤5 cm with advanced local features	LN1 – Ipsilateral peribronchial, perihilar, or intrapulmonary lymph node involvement	M0
Stage IIIA	T3/T4 – Tumor size >5 cm or invasion into adjacent structures	LN2 – Ipsilateral mediastinal or subcarinal lymph node involvement	M0
Stage IIIB/IIIC	T3/T4 – Variable tumor size with advanced local invasion	LN3 – Contralateral mediastinal, hilar, or supraclavicular lymph node involvement	M0
Stage IVA	Any tumor size (TU1–TU4)	Any lymph node involvement	DM1 – Malignant pleural/pericardial effusion or separate contralateral lung nodule
Stage IVB	Any tumor size (TU1–TU4)	Any lymph node involvement	DM2 – Distant metastasis to other organs

## Methodology

### Overview of the ReViT-RF framework

The proposed ReViT-RF framework combines ResNet-50 (a Vision Transformer (ViT)) and radiomic feature fusion modules for conducting in-depth analysis of lung tumors from CT scans. This framework involves five stages: CT image pre-processing, hybrid deep feature extraction, radiomic feature integration, prediction, and explainability analysis. CT scans are first subjected to intensity normalization and CLAHE to enhance the image quality and improve the visibility of the tumors. The ResNet-50 backbone is used to extract discriminative local features of the tumor involving shape, texture, and structural information, while the Vision Transformer is responsible for capturing global spatial dependencies through self-attention mechanisms. These deep features are then combined with radiomic features such as size, shape, texture and spatial features to construct a clinically relevant feature representation. The fused representation is subsequently used for the classification of benign–malignant and for independent prediction of TNM components (T, N, and M). Lastly, Grad-CAM visualizations provide interpretability and clinical transparency to the proposed framework, revealing the tumor regions contributing to the model predictions.

### Hybrid ResNet-50 and vision transformer feature learning

The proposed ReViT-RF method combines ResNet-50-based convolutional feature learning with Vision Transformer (ViT)-based global contextual representation learning to enable accurate lung tumor analysis from CT images. The conventional CNNs can successfully capture local visual patterns, such as tumor textures, contour features, shape variations, and morphology features. Yet, they can only model long-range spatial relationships between different tumor regions to a limited degree. To address this challenge, the proposed framework employs a Vision Transformer module using self-attention techniques to capture global dependencies and context relationships in CT images. In the ReViT-RF framework, the CT images are concurrently processed by two different feature extraction streams. The ResNet-50 branch learns discriminative local tumor representations and the Vision Transformer branch learns global anatomical and spatial information. Both networks were able to extract features that gave a comprehensive representation of the lung nodules, which led to better benign–malignant classification and accurate prediction of the T, N, and M components of TNM staging.

The preprocessed CT images are then used to extract local, tumour-specific features by using ResNet-50 as its convolutional backbone. It is an efficient residual learning approach that is capable of learning hierarchical feature representation and solving the degradation issue of deep neural networks. The CT image is augmented by ${\text{X}}_{\text{i},\text{j}}^{\text{aug}}$ and then input into a ResNet-50 network to get the convolutional feature representation:


$$\begin{array}{c} F_{i}^{CNN}=f_{ResNet\text{5}\text{0}}(X_{i,j}^{aug}) \end{array}$$ (8)

Where$\;{\text{F}}_{\text{i}}^{\text{CNN}}$ represents the extracted CNN feature vector containing discriminative information related to tumor texture, shape, intensity variations, and morphological patterns. These learned features are further utilized for preliminary benign–malignant classification. The probability of malignancy is estimated using a fully connected classification layer, as given below:


$$\begin{array}{c} \tilde{y_{i}}=\sigma (W_{b}F_{i}^{CNN}+b_{b}) \end{array}$$ (9)

Where ${\text{W}}_{\text{b}}$ and b represent the learnable weights and bias parameters of the classification layer, respectively, and σ(·) denotes the sigmoid activation function that generates the malignancy probability score.

The ResNet-50 classification branch is trained using the Binary Cross-Entropy (BCE) loss function:


$$\begin{array}{c} L_{BCE}=-\frac{\text{1}}{N}\sum_{i=\text{1}}^{N}{[y}_{i}log\left(\tilde{y_{i}}\right)-(\text{1}+y_{i})\text{log}(\text{1}-\tilde{y_{i}})) \end{array}$$ (10)

Where ${\text{y}}_{\text{i}}$ signifies the ground-truth malignancy label and $\tilde{{\text{y}}_{\text{i}}}$ signifies the predicted probability, minimizing the binary cross-entropy (BCE) loss enables the network to learn robust tumor representations and improve discrimination between benign and malignant nodules. The features extracted by ResNet-50 are subsequently combined with Vision Transformer embeddings and radiomic descriptors using the proposed feature fusion approach, enabling comprehensive prediction of TNM components.

For a resized CT image ${\text{X}}_{\text{i}}^{\text{res}}$, the Vision Transformer extracts global feature representations as follows:


$$\begin{array}{c} F_{i}^{ViT}=T_{ViT}(X_{i}^{res}) \end{array}$$ (11)

Where ${\text{T}}_{\text{ViT}}$ denotes the Vision Transformer encoder, and ${\text{F}}_{\text{i}}^{\text{ViT}}$ represents the extracted global feature representation. These features contain contextual information related to tumor location, spatial distribution, and inter-regional dependencies, which may not be effectively captured by conventional convolutional operations.

The MHSA process, which dynamically allocates importance weights to various picture patches, is the central function of the Vision Transformer. The following is the definition of the scaled dot-product attention mechanism:


$$\begin{array}{c} Attention(Q,K,V)=Softmax\left(\frac{{QK}^{T}}{\sqrt{d_{k}}}\right)V \end{array}$$ (12)

Where Q, K, and V denote the query, key, and value matrices generated from the patch embeddings, respectively, and ${\text{d}}_{\text{k}}$ represents the dimensionality of the key vectors. The attention mechanism enables the model to identify significant relationships among tumor regions by assigning higher weights to clinically relevant image patches.

### Radiomic and GLCM feature extraction

Medical image processing for extracting radiomic features is a process aimed at mapping medical images into high-dimensional quantitative descriptors that objectively characterize tumor phenotypes. Unlike deep learning features, radiomic features are handcrafted and clinically interpretable, enabling the evaluation of tumor morphology, intensity distribution, and intratumoral texture heterogeneity. The proposed ReViT-RF framework utilizes deep features extracted from the ResNet-50 and Vision Transformer branches and integrates radiomic features as quantitative biomarkers that are closely associated with tumor malignancy and progression.

Initially, the lung nodule region of interest (ROI) is obtained through preprocessing and segmentation. Let the segmented lung nodule region be denoted as:


$$\begin{array}{c} \Omega =\left\{(x,y)|M(x,y)=\text{1}\right\}, \end{array}$$ (13)

Where  M(x,y) denotes the binary segmentation mask, and Ω contains all pixels belonging to the pulmonary nodule.

The radiomic feature extraction module computes a comprehensive feature vector from the segmented region of interest (ROI).


$$\begin{array}{c} f_{G}=F_{G}(\Omega )=\left[f_{shape},f_{first-order},f_{GLCM}\right] \end{array}$$ (14)

Where ${\text{f}}_{\text{shape}}$, ${\text{f}}_{\text{first}-\text{order}}$, and ${\text{f}}_{\text{GLCM}}$ represent the shape, intensity, and texture descriptors, respectively.

Shape descriptors quantify the geometric characteristics of pulmonary nodules. One important descriptor is compactness, which measures the extent to which a pulmonary nodule resembles a circular shape.


$$\begin{array}{c} C=\frac{\text{4}\pi A}{P_{\text{2}}} \end{array}$$ (15)

Where A indicates the segmented tumor area and P indicates the boundary perimeter. Higher compactness generally represents a smoother lesion, whereas irregular malignant nodules typically exhibit lower compactness.

The distribution of voxel intensity values is described using first-order statistics, which do not consider the spatial relationships among neighboring voxels. The mean intensity is calculated as follows:


$$\begin{array}{c} \mu =\frac{\text{1}}{N}\sum_{i=\text{1}}^{N}I_{i} \end{array}$$ (16)

Where ${\text{I}}_{\text{i}}$ represents the intensity of the ${\text{i}}^{\text{th}}$ voxel, and N is the total number of voxels within the ROI.

Likewise, the intensity variation is measured using the standard deviation.


$$\begin{array}{c} \sigma =\sqrt{\frac{\text{1}}{N}\sum_{i=\text{1}}^{N}{\left(I_{i}-\mu \right)}^{\text{2}}} \end{array}$$ (17)

These statistical descriptors capture the average density and heterogeneity of pulmonary lesions.

To capture spatial texture information, the GLCM is computed. The normalized GLCM is defined as follows:


$$\begin{array}{c} P(i,j)=\frac{C(i,j)}{\sum_{i}\sum_{j}C(i,j)} \end{array}$$ (18)

Where C(i,j) indicates the number of neighboring pixel pairs with gray levels i and j for a predefined distance and orientation. The normalized matrix represents the probability of occurrence of each gray-level pair.

Among the various GLCM descriptors, contrast measures the local intensity variation and is calculated as follows:


$$\begin{array}{c} Contrast=\sum_{i}\sum_{i}\left(i-j{)}^{\text{2}}P(i,j\right) \end{array}$$ (19)

Large contrast values indicate greater texture irregularities, which are commonly observed in malignant tumors.

Another important descriptor is homogeneity, which measures the similarity between neighboring gray-level intensities.


$$\begin{array}{c} Homogeneity=\sum_{i}\sum_{j}\frac{P(i,j)}{\text{1}+{\left(i-j\right)}^{\text{2}}} \end{array}$$ (20)

Higher homogeneity values indicate smoother and more uniform tissue structures, whereas heterogeneous tumors typically exhibit lower homogeneity values.

The final radiomic feature vector is created by concatenating all of the extracted radiomic descriptors.


$$\begin{array}{c} f_{G}=f_{shape};f_{first\;order};f_{GLCM} \end{array}$$ (21)

The resulting radiomic feature vector captures the quantitative aspects of the morphology, intensity distribution and texture heterogeneity of pulmonary nodules. The handcrafted features carry meaningful information which complements the features learned through automatic learning by CNN and Vision Transformer, providing clinically meaningful information. The proposed ReViT-RF framework not only achieves thorough multimodal feature learning to enhance the prediction of T, N, and M stage components but also benefits from the fused feature representation, allowing it to successfully classify lung nodules as benign or malignant.

### Multimodal feature fusion

The proposed ReViT-RF framework incorporates three complementary feature representations, which form a comprehensive characterization of pulmonary nodules. ResNet-50 branch detects local morphological and structural features such as nodule boundaries, intra-nodular intensity variations and fine-grained texture patterns. The Vision Transformer branch is based on the self-attention mechanism that captures global contextual information and long-range spatial information of the image. In parallel, the radiomic branch quantifies tumor characteristics, such as shape descriptors, first-order intensity statistics and texture heterogeneity features including Gray-Level Co-occurrence Matrix (GLCM) features. These representations are complementary and describe pulmonary nodules in three different ways: local deep features, global contextual features and handcrafted quantitative features. The combination of these multiple representations increases feature discrimination, boosts model robustness and leads to better diagnostic performance.

For an input CT image x, the three feature extraction branches generate the following feature representations:


$$\begin{array}{c} f_{R}=F_{R}\left(x;\theta_{R}\right) \end{array}$$ (22)

$$\begin{array}{c} f_{V}=F_{V}(x;\theta_{V}) \end{array}$$ (23)

$$\begin{array}{c} f_{G}=F_{G}(x) \end{array}$$ (24)

Where ${\;f}_{R}(.)$, $f_{V}\;(.)$ and $f_{G}(.)$ denote the ResNet-50, Vision Transformer, and radiomic feature extraction functions, respectively. Here, $\theta_{R}$and $\theta_{V}$ represent the trainable parameters of the deep learning branches, whereas the radiomic features are extracted using predefined image descriptors.

Since the three feature vectors have different dimensionalities and statistical distributions, each feature vector is projected into a common d-dimensional latent space using a linear projection followed by layer normalization:


$$\begin{array}{c} z_{k}=LN\left(W_{k}f_{k}+b_{k}\right)\;k\in \{R,V,G\} \end{array}$$ (25)

Where $W_{k}$ and $b_{k}$ denote the learnable projection matrix and bias vector, respectively, and LN (.) represents layer normalization. This transformation aligns the feature distributions, preventing any modality with higher dimensionality or greater numerical magnitude from dominating the fusion process.

To adaptively determine the contribution of each modality, learnable importance weights are estimated using the Softmax function as follows:


$$\begin{array}{c} \alpha_{k}=\frac{\text{exp}(s_{k})}{\sum_{j\in \{R,V,G\}}\text{exp}(S_{j})}\;k\in \{R,V,G\} \end{array}$$ (26)

Subject to


$$\begin{array}{c} \sum_{k\in \{R,V,G\}}\alpha_{k}=\text{1} \end{array}$$ (27)

Where $s_{k}$ denotes the trainable relevance score corresponding to the $k^{th}$ feature branch. Higher relevance scores indicate greater importance of the corresponding modality during feature fusion.

The weighted feature vectors are concatenated to generate a multimodal representation.


$$\begin{array}{c} z_{F}=[\alpha_{R}z_{R};\alpha_{V}z_{V};\alpha_{G}z_{G}] \end{array}$$ (28)

Where [.;.} signifies feature concatenation.

A fully linked fusion layer with a nonlinear activation function and dropout regularisation is then employed to enhance the fused representation.


$$\begin{array}{c} h_{F}=Dropout(ReLU(W_{F}z_{F}+b_{F})) \end{array}$$ (29)

Where ${\text{W}}_{\text{F}}$ and ${\text{b}}_{\text{F}}$ are the trainable parameters of the fusion layer, and ${\text{h}}_{\text{F}}$ denotes the final multimodal embedding.

The proposed weighted intermediate fusion strategy preserves modality-specific information while simultaneously learning complementary interactions among local CNN features, global Transformer representations, and clinically interpretable radiomic descriptors. The learned multimodal embedding, $h_{F}$, serves as a shared feature representation for both the benign–malignant classification network and the independent T-stage, N-stage, and M-stage prediction heads. This unified representation enables accurate tumor diagnosis and precise TNM staging within a single end-to-end learning framework.

### Prediction framework

#### Tumor classification (Benign vs Malignant).

The suggested model performs binary classification of lung nodules into two classes, namely benign and malignant, based on the feature representation obtained through the ReViT-RF model. The combined feature representation is fed into a fully connected classification layer that uses the sigmoid function as the activation function. The malignancy probability is then estimated, and based on the predefined decision threshold, the nodule is classified as either benign or malignant.

### TNM staging system (multiclass classification)

Additional information beyond a mere benign–malignant classification is needed to accurately assess tumor progression. In order to give a comprehensive assessment of lung cancer development, the proposed ReViT-RF is suggested to predict the three primary components of TNM. This method is able to provide a more transparent and clinically relevant assessment, as it delivers component-level predictions instead of setting a pre-defined disease stage. The transformer-based feature refinement module generates an optimized fused feature representation (H_i_) that is passed to three independent prediction branches. The individual branches are taught the distinctive features of each component of the TNM.

The output $H_{i}$ is then used to predict the TNM components using separate softmax layers.


$$\begin{array}{c} {\hat{T}}_{i}=softmax(W_{T}H_{i}+b_{T}) \end{array}$$ (30)

$$\begin{array}{c} {\hat{N}}_{i}=softmax(W_{N}H_{i}+b_{N}) \end{array}$$ (31)

$$\begin{array}{c} {\hat{M}}_{i}=softmax(W_{M}H_{i}+b_{M}) \end{array}$$ (32)

Equation (30) represents the tumor size, Equation (31) represents lymph node involvement, and Equation (32) represents metastasis. The overall loss is calculated as the sum of the categorical cross-entropy losses of these three components, as expressed in Equation (33).


$$\begin{array}{c} L_{TNM}=L_{T}+L_{N}+L_{M} \end{array}$$ (33)

Following the predictions of the T, N, and M components, the overall stage is determined by combining their respective predictions according to the TNM criteria provided in Table 1. The predicted values of T, N, and M are mapped to the corresponding overall stage based on established staging rules, eliminating the need for an additional neural network-based classification branch. This approach ensures that each component is independently predicted while simultaneously generating an imaging-based overall stage prediction. The resulting stage provides an imaging-assisted estimation of the staging process; however, it does not replace clinically validated pathological TNM staging.

### Explainability using Grad-CAM

Although deep learning models frequently produce excellent predictions, their intricate decision-making procedures make them opaque. Finding the areas that affect model predictions in medical image analysis is crucial for increasing clinical acceptance and confidence. In order to visualise the key tumour locations influencing the model’s predictions, the suggested ReViT-RF framework integrates Grad-CAM as an explainability mechanism. Grad-CAM analyses the gradients passing through the final convolutional feature maps of the ResNet-50 branch to create class-specific activation maps. The produced heatmaps show the discriminative areas in CT scans that have a major impact on TNM component prediction and benign–malignant classification.

Equation (34) describes the computation of the Grad-CAM heatmap for a given class c.


$$\begin{array}{c} L_{GRAD-CAM}^{c}=ReLU\left(\sum_{k}\alpha_{k}^{c}A^{k}\right) \end{array}$$ (34)

Where ${\text{A}}^{\text{k}}$ represents the activation map obtained from the convolutional layer, and $\alpha_{\text{k}}^{\text{c}}$ denotes the gradient-based weight that indicates the importance of feature map k for class c.

The Grad-CAM importance weights are computed by propagating gradients to the final convolutional layer.


$$\begin{array}{c} \alpha_{k}^{c}=\frac{\text{1}}{Z}\sum_{u}\sum_{v}\frac{\partial y^{c}}{\partial A_{uv}^{k}} \end{array}$$ (35)

Where Z signifies the total number of pixels in the feature map.

### Localization validation

To quantitatively assess the reliability of the Grad-CAM explanations, the predicted activation regions are compared with expert-annotated tumor regions using two widely adopted localization metrics: Intersection over Union (IoU) and Pointing Game Accuracy (PGA). These metrics evaluate whether the model accurately localizes clinically relevant pulmonary nodules rather than focusing on irrelevant image structures.

The IoU quantifies the degree of overlap between the thresholded Grad-CAM activation region and the ground-truth tumor segmentation mask. It is defined as follows:


$$\begin{array}{c} IoU=\frac{\left|M_{CAM}{\cap M}_{GT}\right|}{\left|M_{CAM}{\cup M}_{GT}\right|} \end{array}$$ (36)

Where $M_{CAM}$ represents the Grad-CAM activation mask and $M_{GT}$ denotes the radiologist-annotated ground-truth tumor mask. An IoU value closer to 1 indicates stronger localization agreement between the model’s explanation and the actual lesion.

The PGA evaluates whether the location of the maximum Grad-CAM activation lies within the annotated tumor region. It is computed as follows:


$$\begin{array}{c} PGA=\frac{\text{1}}{N}\sum_{i=\text{1}}^{N}I(p_{i}^{max}\in M_{GT,i}) \end{array}$$ (37)

Where N represents the total number of test images, $p_{i}^{max}$ indicates the pixel corresponding to the highest Grad-CAM activation for the $I^{th}$ image, $M_{GT,i}$ represents the corresponding ground-truth tumor mask, and I(·) denotes the indicator function that returns 1 if the maximum activation point lies within the annotated lesion and 0 otherwise.

Radiologist validation is additionally performed by visually correlating the generated Grad-CAM heatmaps with expert-annotated tumor boundaries. The combination of IoU, PGA, and expert qualitative assessment provides a comprehensive evaluation of the explainability module, demonstrating that the proposed ReViT-RF technique accurately focuses on clinically relevant pulmonary nodule regions while performing benign–malignant classification and TNM stage prediction.

### Training phase

The suggested ReViT-RF model is trained using a two-phase learning procedure. First, using labelled samples and Binary Cross-Entropy loss, a binary classification model is trained to classify the CT samples as either benign or malignant. The model is trained to identify discriminative features during this phase, and validation data is supplied for the hyperparameter optimisation and performance evaluation. The TNM staging is carried out in a multiclass classification framework, where the deep features extracted from ResNet-50 and Vision Transformer (ViT) are combined with handcrafted radiomic features obtained from GLCM and anatomical localization information after the identification of the presence of malignant tumors. Optimization of the fused feature representation is performed with categorical cross-entropy loss and the SoftMax activation is used to predict each TNM category. Confidence-weighted majority voting is applied to get the patient-level staging results from the slices, so as to give reliable and leakage-free evaluation.

The ReViT-RF model first predicts at each slice then aggregates the predictions for the final patient level classification. The whole process is shown in Fig 2.

**Fig 2:**
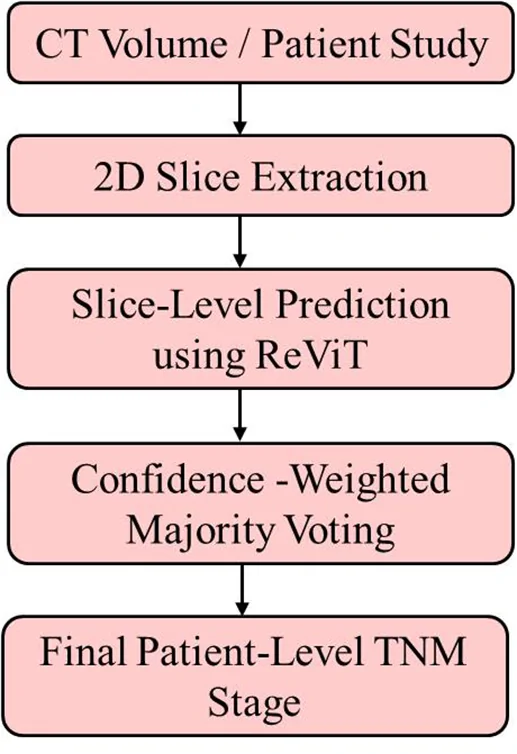
Slice-to-patient aggregation workflow.

The final patient-level TNM stage is determined by aggregating slice-level predictions using confidence-weighted majority voting, ensuring that the model incorporates information from all relevant slices.

### Testing phase

To improve the multiclass classification task’s interpretability, the suggested model pipeline makes use of Explainable Artificial Intelligence (XAI) approaches. For each TNM category, Grad-CAM is utilised to determine the most significant areas in CT scans. This explainability mechanism improves the decision-making process’s transparency, dependability, and therapeutic relevance. The suggested model’s performance and robustness are assessed using F1-score, AUC-ROC, accuracy, precision, recall, and specificity. Both general and class-specific system validation of the suggested system based on the expected result is made possible by this thorough evaluation method.

## Result

### Experimental setup

Intensity normalization and CLAHE were applied to all CT images for their intensity normalization and enhanced contrast, respectively, to provide better visualization of the tumors. The output images were resized to 224 × 224 × 3 pixels to align with the input size of ResNet-50 and ViT. Random rotation, horizontal flip, scaling and zooming techniques were applied to the training data to enhance model robustness and prevent overfitting. The proposed ReViT-RF architecture successfully captures local morphological and textural features of CT images, in addition to capturing long-range contextual dependencies by combining ResNet-50 and a Vision Transformer (ViT). Meanwhile, handcrafted radiomic descriptors (shape, size, first-order intensity, texture and spatial) were extracted and combined with the deep feature representations. The multimodal feature fusion strategy aimed to enhance the classification of lung tumor and component-wise T, N, M prediction for benign and malignant lung tumors. The benign-malign task was optimized using binary cross-entropy loss and predictions of T, N and M were optimized separately using categorical cross-entropy loss. The model’s performance was assessed using stratified 10-fold cross-validation, and data leakage was avoided by keeping training and test patients distinct at the patient level. The evaluation metrics were accuracy, precision, recall, F1 score, specificity, Matthews correlation coefficient (MCC), area under the receiver operating characteristic curve (AUC-ROC), and confusion matrix. Additionally, by employing Grad-CAM to evaluate the tumour regions that generated the model’s predictions, the proposed framework’s interpretability and clinical utility were enhanced.

Python 3.10, PyTorch 2.1, and other libraries like NumPy, OpenCV, scikit-learn, and Matplotlib were used to create the suggested framework. Each investigation was carried out on a workstation running Ubuntu 22.04, an Intel Core i7 processor with 32 GB of RAM, and an NVIDIA RTX 3080 GPU with 10 GB of RAM. The AdamW optimiser was used to optimise the network with a batch size of 32, 60 training epochs, and an initial learning rate of 1 × 10^−2^. To ensure a fair comparison, all baseline and suggested models were trained with the identical hyperparameters. Using the same hardware and software configuration, the computational efficiency was also assessed by measuring the number of floating point operations (FLOPs), average inference time, GPU memory utilisation, and trainable parameters.

### Results obtained by ReViT-RF model

A representative lung CT slice is presented for comparing the different three preprocessing schemes provided in Fig 3. The original CT image is naturally represented in gray levels but for some area, the contrast is low. z-score standardization is used to normalize the image by shifting the intensity values of the pixels to the mean and standard deviation of the image. It translates pixel values to a common scale, harmonizes the global contrast and enhances the image for machine learning-based analysis. The third image displayed is the CLAHE-enhanced image, which is an image obtained using the contrast-limited adaptive histogram equalization to improve local contrast in small local regions (tiles) while minimizing noise amplification. The method effectively enhances the detectability of fine structures, including pulmonary nodules and blood vessels, which facilitates improved interpretation and automated image analysis. In general, each of these methods has a different purpose: The original image is used to store raw CT information, the normalization image is used to normalize the input information for the model, and the CLAHE image is used to enhance the local features to improve the visualization and extraction of the features.

**Fig 3:**
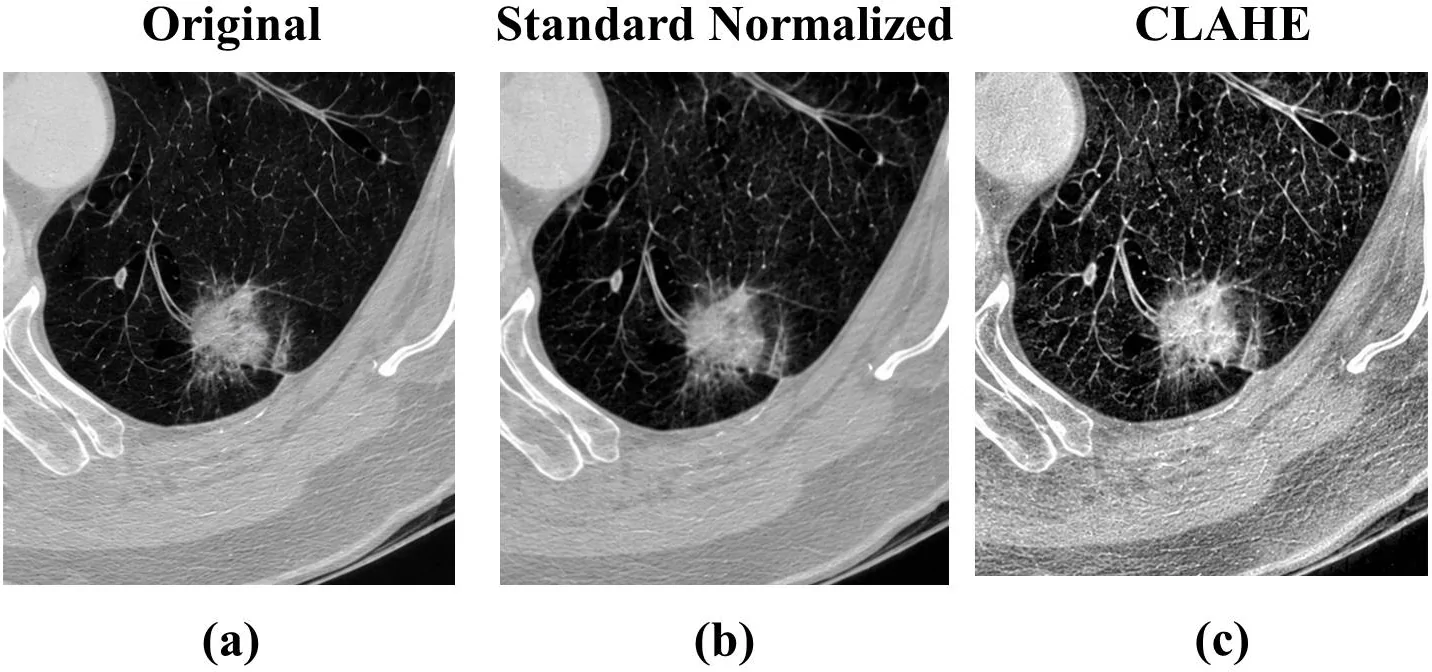
Preprocessing steps. From left to right: (a) Original CT slice, (b) after intensity standard normalization, and (c) after CLAHE enhancement. Standard normalization rescales the voxel intensities to a fixed range, reducing inter-scan variability, while CLAHE improves local contrast and enhances the visibility of the lung nodule and surrounding structures.

To apply the data augmentation techniques presented in Fig 4, the image in the CT scan of the lung is used. The original slice is shown on the far left, and five rotations, scaling, flips, intensity changes and elastic deformations are shown on the right. These methods are used to artificially expand the size of the data set and add in realistic variations, to make the model more generalizable. The model can learn to recognize the important features of lung nodules and their surrounding tissues in different variations of the images and detect these features in images that have been rotated, zoomed, or otherwise altered. This is especially useful in the field of medical imaging, where precise measurements and predictable predictions are vital to clinical decision-making. The images in the Augmented column will show different views of the same anatomical region, but in anatomical consistency.

**Fig 4:**
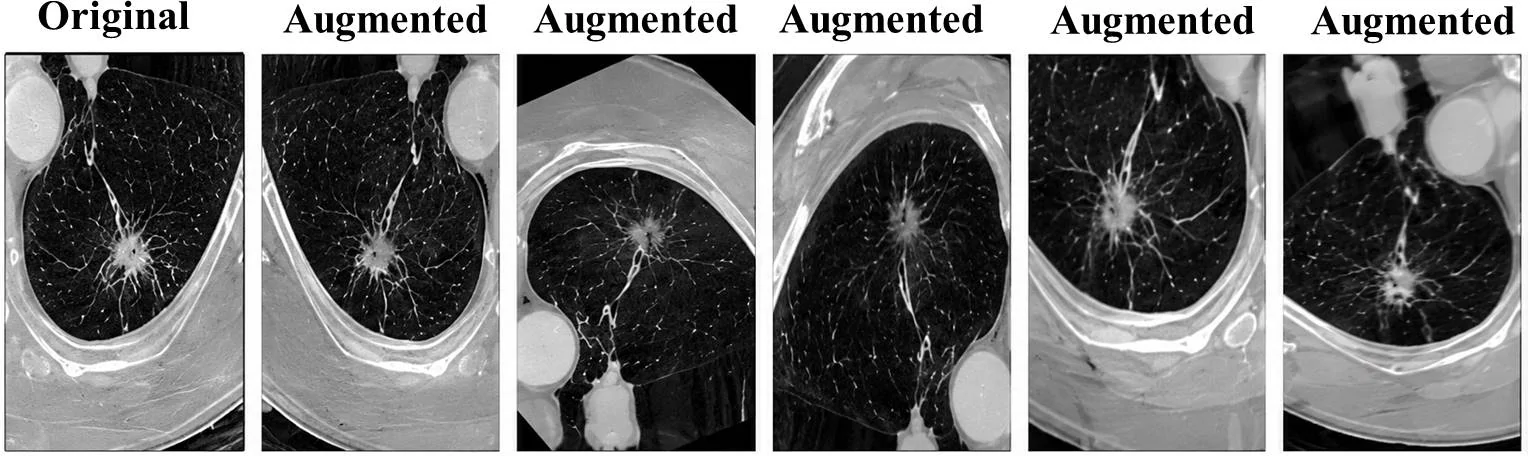
Samples of original and augmented images.

### Explainability and localization performance

Fig 5 presents the prediction and visualization of malignant regions in lung CT scan slices using Grad-CAM. The left image, labelled Non-Cancer, shows no highlighted abnormal regions, indicating that the model has classified the lungs as healthy. In contrast, the cancerous CT slices—Slice 2, Slice 3, and Slice 4—display affected regions highlighted through Grad-CAM heatmaps, with yellow boundaries and red activation areas. These highlighted regions represent the image features that most strongly influenced the model’s identification of malignant structures. Grad-CAM improves the interpretability and spatial comprehension of the model’s predictions by precisely localising tumor-related regions using gradient information from the last convolutional layer. Slice 2, Slice 3, and Slice 4 correspond to Stage 2, Stage 2, and Stage 1, respectively, and demonstrate noticeable variations in the size, location, and intensity of the activated regions. Overall, the results confirm the effectiveness of explainable artificial intelligence in medical imaging, as the model predictions correspond with clinically relevant tumour locations and enhance the transparency and interpretability of the diagnostic process.

**Fig 5:**
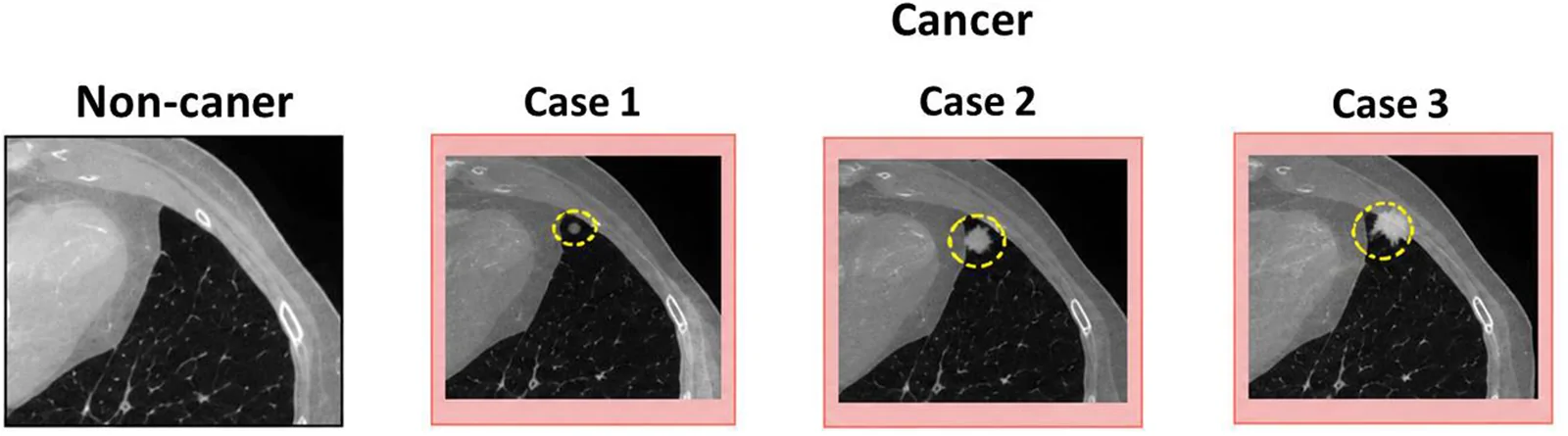
Illustration of data labeling with TNM staging with Grad-CAM mapping. Left: non-cancer (benign) CT slice with no tumor. Right: cancer (malignant) cases labeled according to TNM staging. Stage 1 represents a small, localized tumor; Stage 2 indicates a larger tumor with possible local spread; and Stage 3 represents an even larger tumor with more extensive local involvement. Grad-CAM heatmaps (yellow dashed circles) indicate the image regions that the model focuses on during decision-making.

The overlap between the expert-annotated nodule mask and the thresholded Grad-CAM region was assessed using the Intersection over Union (IoU) metric. A higher IoU value indicates greater spatial agreement between the model-generated explanation and the actual lesion location.

Table 2 shows that malignant and T-component predictions achieved the highest mean IoU values of 0.782 and 0.768, respectively, indicating strong spatial agreement between the Grad-CAM activation regions and the expert-annotated tumor masks. The N- and M-component predictions exhibited slightly lower localization performance, which may be attributed to the limited visibility of lymph node involvement and distant metastases in individual CT slices. The overall mean IoU of 0.747 demonstrates that the proposed ReViT-RF framework consistently focuses on clinically relevant pulmonary lesion regions.

**Table 2:** IoU-based localization performance of the proposed ReViT-RF framework.

Prediction category	Number of images	Mean IoU	Standard deviation
Benign	120	0.714	0.086
Malignant	120	0.782	0.074
T-component prediction	120	0.768	0.079
N-component prediction	120	0.741	0.083
M-component prediction	120	0.729	0.088
Overall	600	0.747	0.083

Pointing Game Accuracy was used to determine whether the pixel with the highest Grad-CAM activation was located within the expert-annotated tumor mask.

Table 3 shows that the proposed ReViT-RF framework achieved a high overall Pointing Game Accuracy of 94.38%, indicating that the maximum Grad-CAM activation was located within the expert-annotated tumour region in most CT images. The benign–malignant classification task achieved the highest localization accuracy of 97.08%, demonstrating that the model consistently focused on the pulmonary nodule during binary diagnosis. The T-component prediction achieved an accuracy of 95.00%, whereas the N- and M-component predictions attained accuracies of 93.33% and 92.08%, respectively. These findings verify that the explainability module successfully locates clinically significant lesion locations, enhancing the suggested ReViT-RF framework’s interpretability and dependability.

**Table 3:** Pointing Game Accuracy of Grad-CAM localization.

Prediction task	Correct localization	Total images	Pointing Game Accuracy (%)
Benign–malignant classification	233	240	97.08
T-component prediction	228	240	95.00
N-component prediction	224	240	93.33
M-component prediction	221	240	92.08
Overall	906	960	94.38

#### TNM component prediction performance.

A binary classification experiment is based on the discrimination between benign and malignant tumors, while the TNM analysis is only applied to malignant cases. The proposed ReViT-RF is able to predict the T, N, and M components independently to characterize the tumor in detail. In contrast to binary classification, TNM prediction implies multi-class, component-wise learning problem, which aims at identifying tumor size characteristics (termed T-stage), lymph node involvement (termed N-stage), and the metastatic status (termed M-stage). The component-wise evaluation is used to show the ability of the proposed framework to give clinically meaningful staging information.

The qualitative evaluation of the Grad-CAM heatmaps is shown in Table 4, which was performed by experienced thoracic radiologists. The results of the 960 assessed CT images showed that 864 cases (90.00%) were correctly localized, with excellent agreement between the regions identified and the annotated pulmonary nodules. Seventy-one cases (7.40%) were classified as partially localized, where the activation regions covered only a portion of the lesion or slightly extended beyond the tumor boundaries. Only 25 cases (2.60%) were identified as incorrectly localized. These findings demonstrate that the proposed ReViT-RF framework enhances interpretability, transparency, and clinical reliability for benign–malignant classification and TNM stage prediction by accurately identifying clinically relevant pulmonary nodule regions.

**Table 4:** Radiologist assessment of Grad-CAM explanations.

Assessment category	Number of cases	Percentage (%)
Correctly localized	864	90.00
Partially localized	71	7.40
Incorrectly localized	25	2.60
Total	960	100.00

### Statistical analysis of ReViT-RF model for sample slices

Rather than at the individual slice level, the statistical analysis was carried out at the patient level. The suggested ReViT-RF model produced prediction probabilities for every CT slice; however, a confidence-weighted majority voting method was used to aggregate the slice-level probabilities relating to the same patient in order to achieve the overall patient-level prediction. As a result, all presented metrics such as accuracy, precision, recall, F1-score, AUC-ROC, confidence intervals, and p-values derived from statistical tests represent patient-level performance.

For the paired t-test analysis using 10-fold patient-level cross-validation, each fold was considered an independent evaluation unit. The obtained result was t(9) = 5.72, p = 0.0003, indicating that the proposed ReViT-RF model achieved statistically significant improvement compared with the baseline approach. Patient-level bootstrapping was applied to estimate the 95% confidence intervals. The high accuracy should be interpreted carefully because the TNM labels were derived from imaging-based proxy annotations rather than from a full pathological TNM ground-truth dataset, even though the non-overlapping confidence intervals show the stability and reliability of the suggested approach.

The six CT scan slices preprocessed using three different methods are shown in Fig 6: Original, Standard Normalization, and CLAHE.

**Fig 6:**
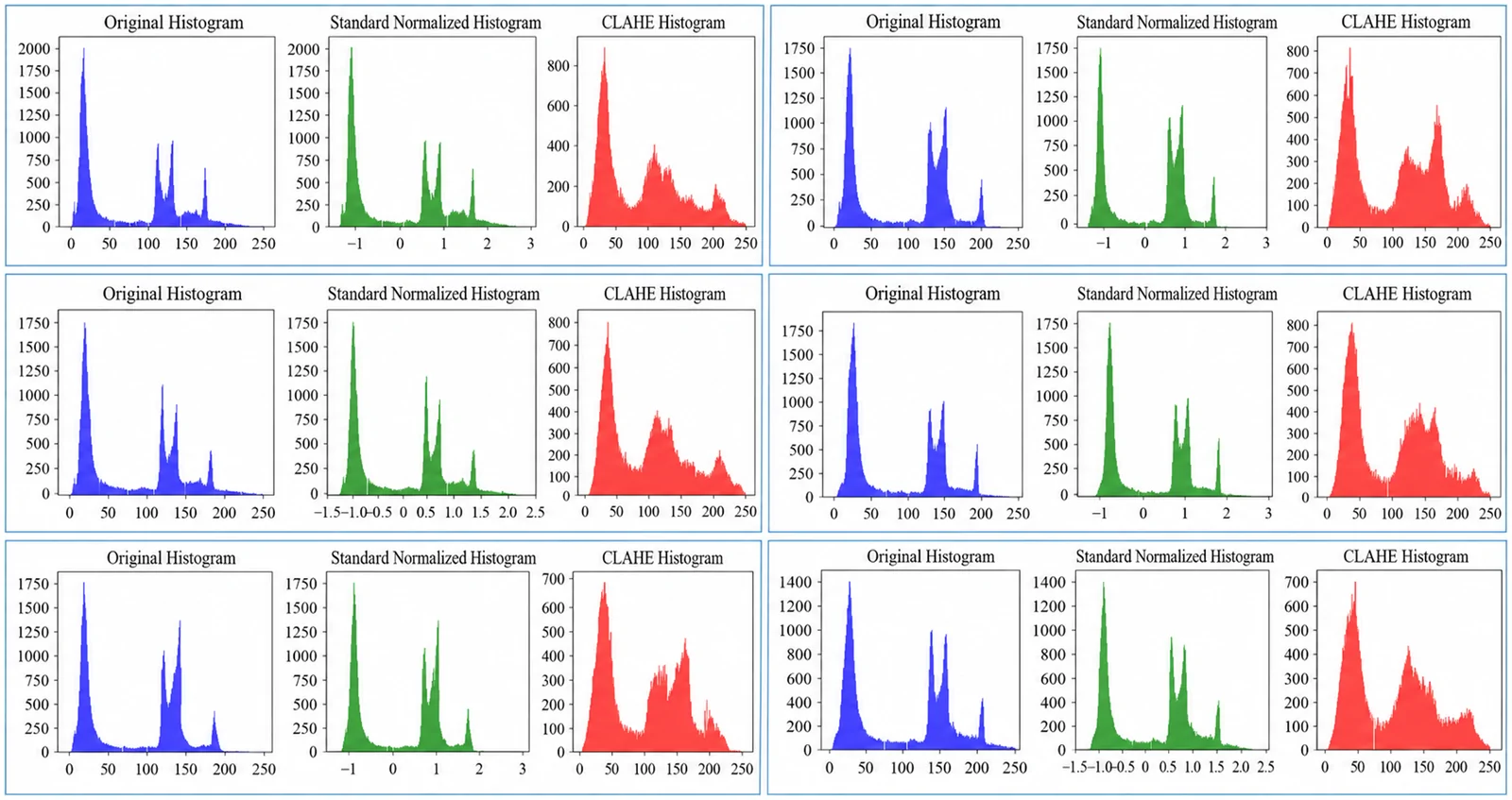
Histogram for sample 6 slices. Red color graph represents CLAHE histogram, Green color graph represent normalized histogram and Blue color graph represent original histogram.

Each row is a CT slice, and each row is a description of the pixel intensity histogram of the CT slice in each method. The blue histograms are the original CT images: the grayscale intensity distributions are plotted. In many of these cases, such as medical imaging, the histograms will have more intensity in the lower intensity regions, associated with background and soft tissue regions.

The green histograms show the standard normalization (zero mean and variance of unity). They are symmetrically distributed about zero and have a bell-shaped distribution. This solution does not necessarily improve the image quality of the images, but it can be advantageous for deep learning and for the numerical stability. The histograms for the CLAHE (red) are, on the other hand, more even. In CT images, CLAHE improves the contrast between subtle details in the image. The curves of the histograms are smoother and more evenly distributed, which suggests that the features are better represented, and can help identify nodules and tissue boundaries, which are crucial for accurate interpretation and improved model performance.

The accuracy, precision, recall, F1 score, and AUC-ROC along with their 95% confidence intervals are presented for the patient performance parameters of the lung cancer classification model in Fig 7. The bar graph indicates the accuracy of the suggested approach on all the evaluation measures, which is good as the accuracy is 97.62%. The consistency and stability of the model predictions on the specified dataset are shown by the error bars, which show the 95% confidence intervals. The confidence intervals, representing the variation in performance, are narrow, and the suggested model is strong, stable and did not change appreciably if the input data were to change.

**Fig 7:**
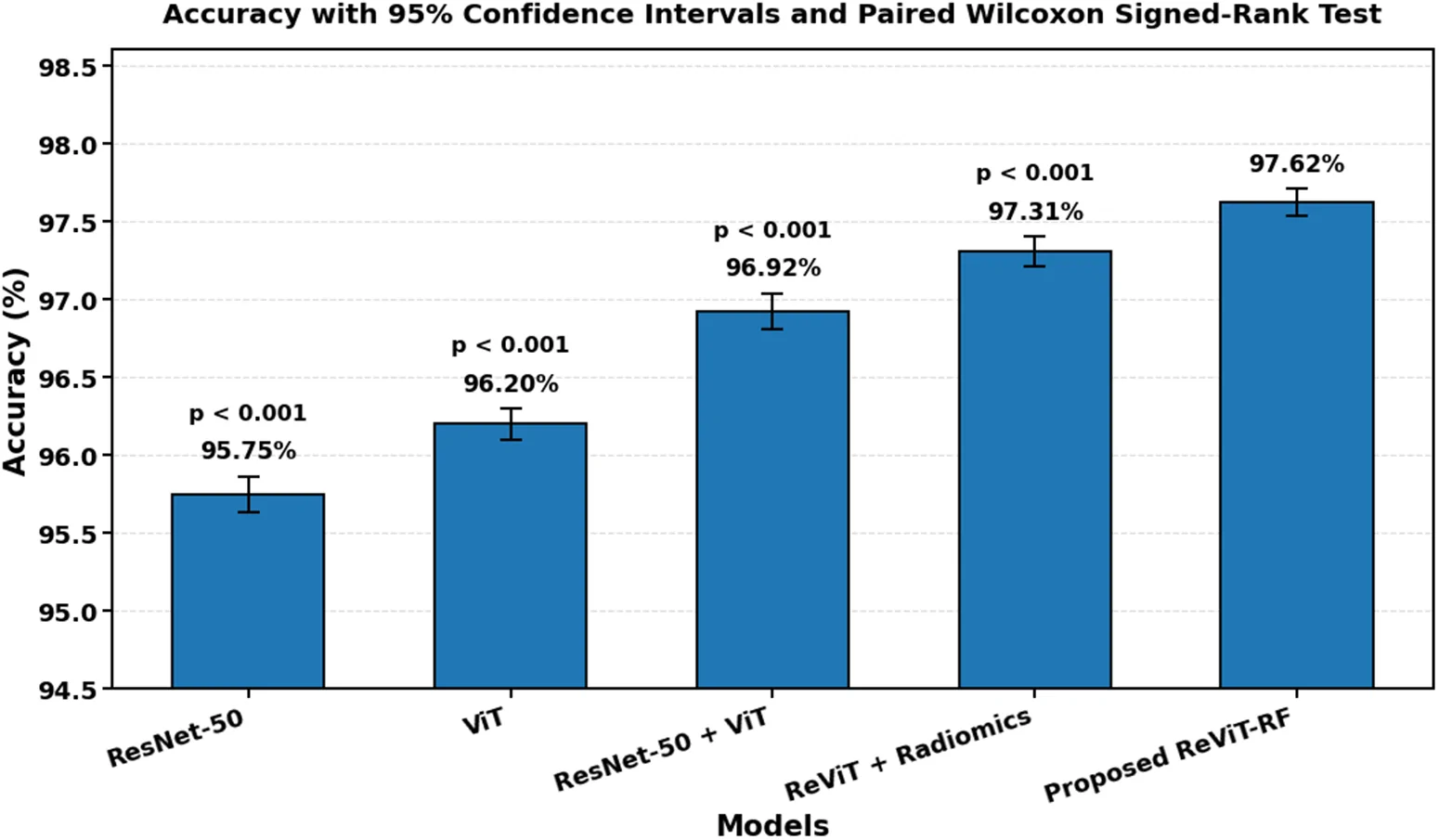
Patient-level performance metrics with 95% confidence intervals.

#### GLCM feature matrix.

Fig 8 displays the GLCM representations of eight sample malignant CT scans, highlighting the textural patterns observed in cancerous regions.

**Fig 8:**
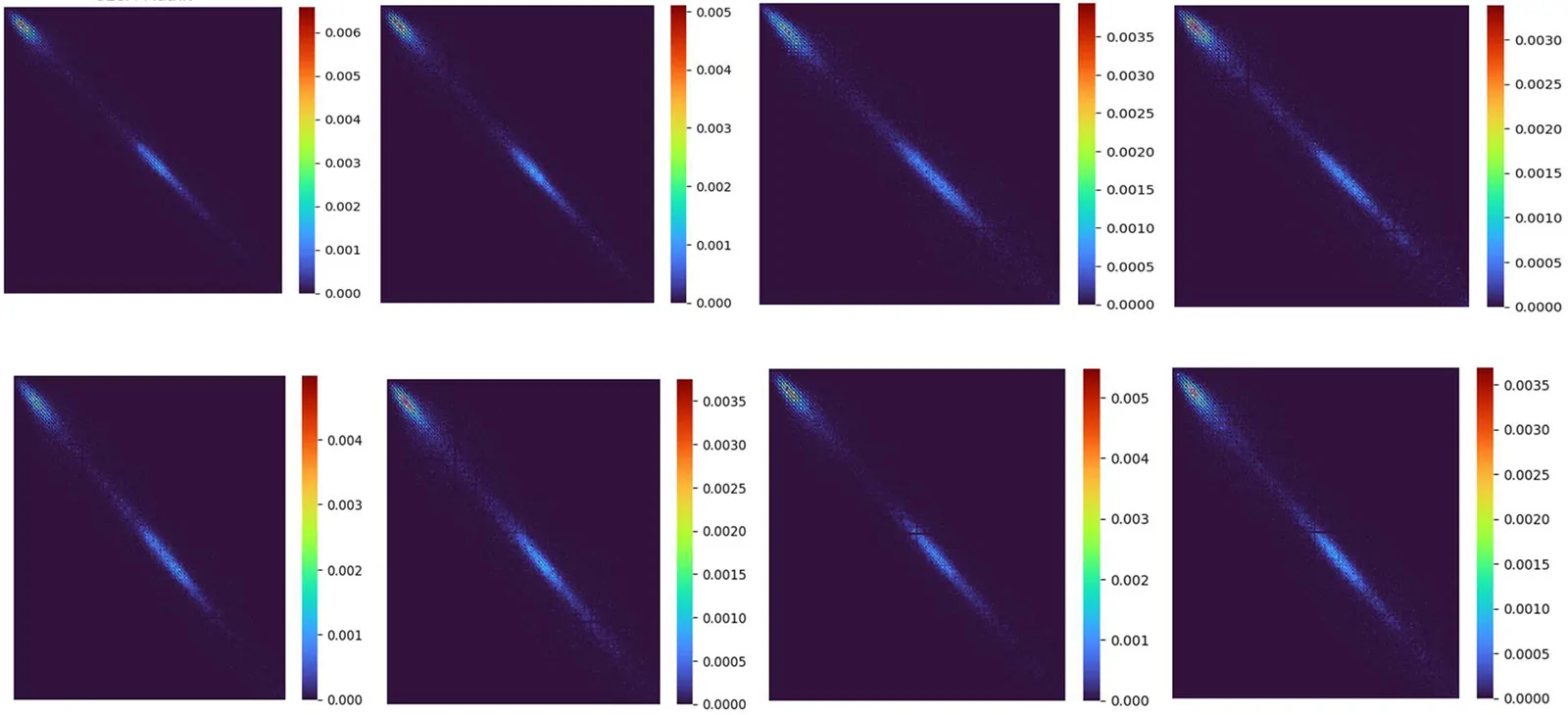
GLCM matrix for sample 8 malignant cases.

A set of normalized GLCMs are computed for each sub-image, which represent the spatial correlation between intensities in a grayscale image. These matrices are often defined by the fact that the values on the diagonal are typically higher, meaning that neighbouring pixels in a homogeneous tissue area are likely to be similar in intensity. Heterogeneity is shown as deviations from the intensity distribution and spread along the diagonals, typically seen in malignant tissues. The following method is used to map colour maps from GLCM: Red areas indicate often occurring gray-level pairs, while blue indicates low probability of occurrence. These differences are an indication of the textural complexity of the samples, possibly related to nodular or irregular structures. GLCMs are also employed to provide quantitative texture features that are crucial to the radiomics-based analysis to distinguish benign from malignant cases, such as energy, homogeneity, correlation, and contrast. The analysis shows that the GLCM techniques is capable of extracting complex textural patterns from CT images of malignant tissues and can be used to provide informative information for diagnostic modelling and classification.

#### Probability of prediction stages.

Fig 9 illustrates the probability distributions of predictions for six CT scan samples obtained using the proposed model. The results demonstrate a high level of confidence in classifying each sample into one of the three TNM cancer stages: Stage 0 (blue), Stage 1 (green), and Stage 2 (red).

**Fig 9:**
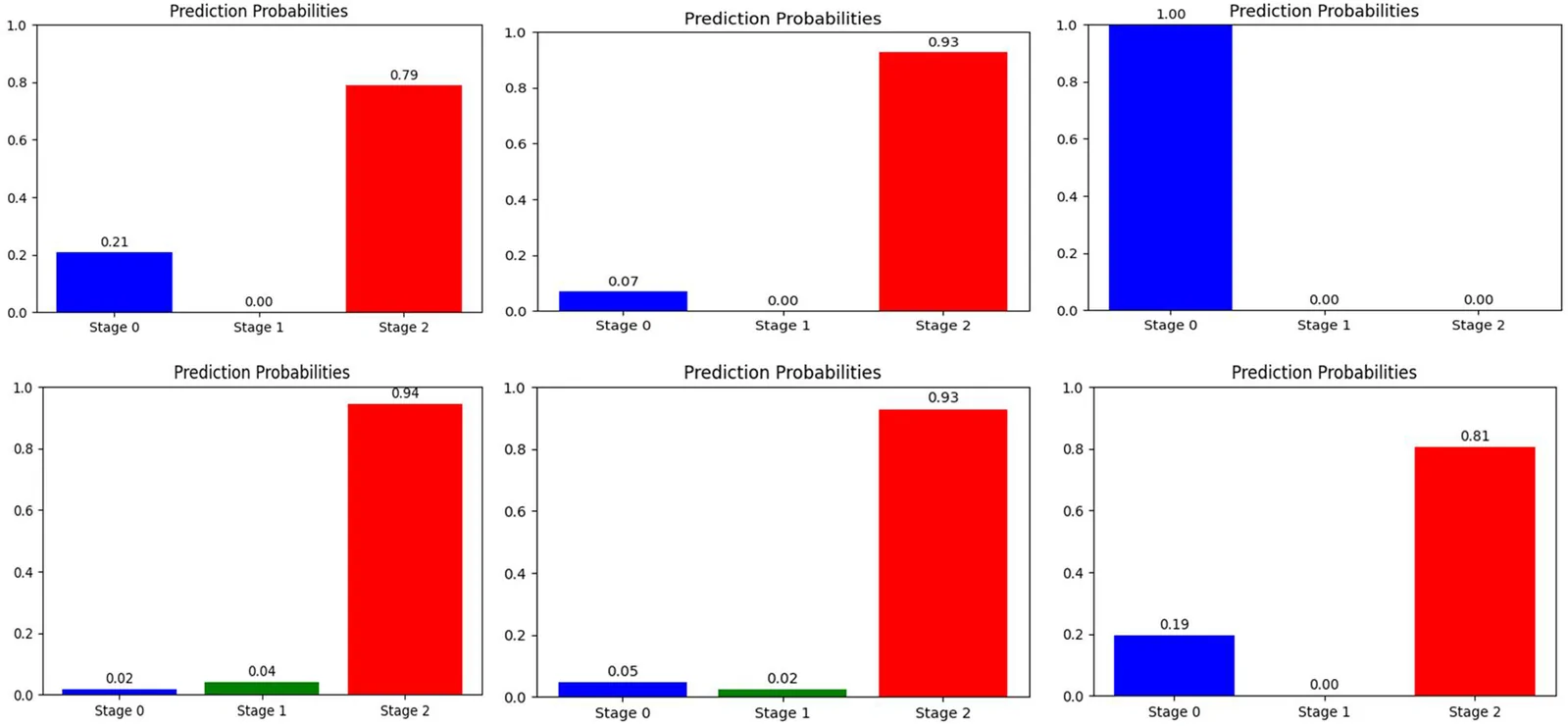
Probability of prediction stages for 6 sample malignant case.

The bar graphs show individual samples with probability values on the y axis (between 0 and 1) and the three stages of the disease on the x axis. Above each of the bars are the values that represent the probability that the model assigns to the stage that is being predicted. There are no predictions in Stage 3 and the predictions in Stage 1 are dominant, with the highest confidence level of 0.93 or 0.94, which is high classification confidence. However, if the probability values are lower (e.g., 0.02 or 0.04) at Stage 0 and 1 sample, this implies that the model is biased towards Stage 2 for some samples. Nonetheless, the accuracy of the model’s predictions some of which reach 100% confidence in Stage 0 cases—is a measure of its performance.

This prediction distribution across the stages of the model shows its ability to distinguish between early and advanced cancer from similar to different stages. Specifically, the high-confidence predictions in Stage 2 have shown the high discriminatory power of the model to identify attributes of aggressive cancer and patterns of high cancer stage in these input CT samples. This visualization allows for a clear understanding of the probability of each cancer stage for each CT sample, giving an interpretable representation which can help with clinical decision making processes.

### Cross validation (K-fold)

The suggested model’s effectiveness, consistency, and robustness are shown in Table 5 using 10-fold cross validation. The model works well and consistently, as evidenced by its classification accuracy, which ranges from 96.54% to 97.62% across all folds. The precision values range from 95.22% to 96.91%, indicating the model’s capacity to reduce false-positive predictions. Recall scores also varied from 94.04% to 96.13%, indicating a rise in sensitivity to genuine positive cases. Additionally, the suggested model offers a compromise between recall and precision with an F1-score ranging from 94.82% to 96.72%. In summary, the findings indicate that the suggested model is resilient, efficient, and stable for classification applications, exhibiting very slight fold-to-fold fluctuations.

**Table 5:** 10-Fold cross validation for ReViT-RF model.

Fold	Accuracy (%)	Precision (%)	Recall (%)	F1-Score
Fold 1	96.54	95.22	94.04	94.82
Fold 2	96.64	95.65	94.37	95.17
Fold 3	96.82	95.84	94.61	95.42
Fold 4	96.91	96.02	94.98	95.67
Fold 5	97.02	96.31	95.25	95.97
Fold 6	97.28	96.42	95.46	96.12
Fold 7	97.34	96.53	95.58	96.22
Fold 8	97.46	96.68	95.75	96.37
Fold 9	97.57	96.71	95.92	96.52
Fold 10	97.62	96.91	96.13	96.72

The data leakage was prevented by 10-fold cross validation at the patient level. To ensure that a patient’s images were not present in both folds, all the CT slices of each patient were allocated to either the training or the evaluation set.

The dataset was split into subgroups of 70% training, 15% validation, and 15% testing to make sure there was no data leakage between the slices and that benign and malignant lung tumours were accurately detected. The CT slices from each patient were constantly positioned in the same partition during the trial. An additional patient-level 10-fold cross validation technique was used to further illustrate the robustness and generalisation capabilities for the analysis of malignant TNM staging. Before being applied to the validation and testing sets, normalisation, CLAHE adaptation, and augmentation strategy parameters were only established for the training set for each fold. Testing and validation data were never used in the model optimisation process. This methodology eliminated information leaks and allowed for a fair and reliable assessment of the model’s performance. The final assessment parameters (accuracy, precision, recall, F1-score, and AUC-ROC) were provided as mean ± standard deviation across all folds in CV and repeated experimental runs for a statistically sound and reproducible performance estimate.

### Epochs for training and testing accuracy

The accuracy of the training and testing is demonstrated with the number of epochs in Fig 10 and it is noted that the accuracy of the model increases during training. The model initially generalizes well with an accuracy of 0.66 at epoch 0, for both training and testing. With increasing number of epochs, the accuracy of both training and testing gradually improves. The testing accuracy in the 20th epoch is 0.71, and the training accuracy is 0.67, indicating that the model starts to acquire more generalized patterns. Both the training and testing accuracies show minor variations in the range of 30−40 epochs; the testing accuracy, however, gradually improves and is maintained at 0.71–0.73, reflecting the continuous improvement of generalization performance. At epoch 50, the training accuracy reaches 0.73 and the testing accuracy reaches 0.79, which shows a significant improvement, with the model learning the features well. The model performs well at epoch 60, with a training accuracy of 0.78 and a testing accuracy of 0.84, showing improved performance in the learning of discriminative features and generalization. The overall results show that the training and testing performance is clearly improved with increasing the number of the training epochs.

**Fig 10:**
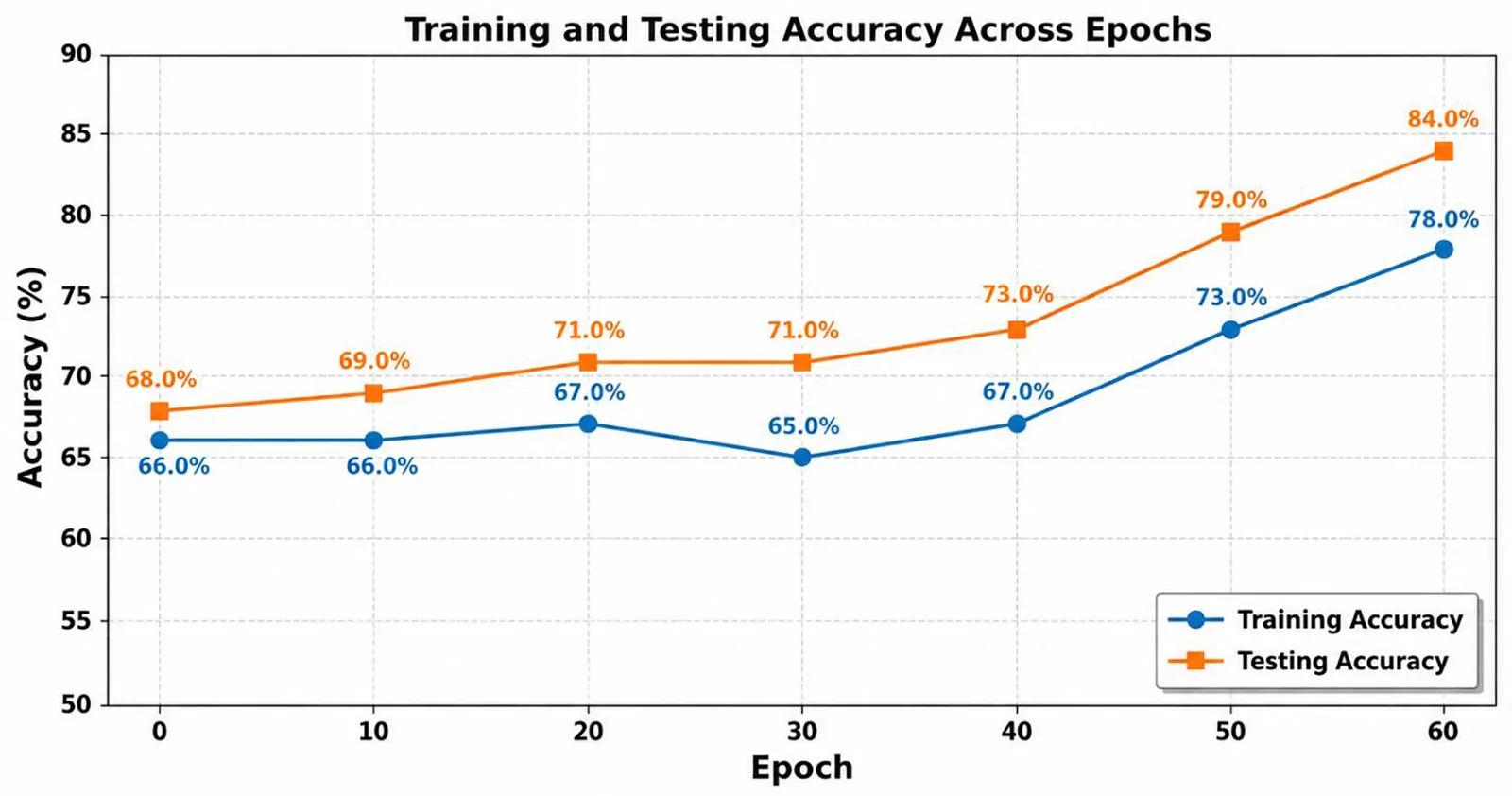
Epochs for training and testing accuracy of ReViT-RF model.

### ROC curve

Fig 11 shows the ROC curve, which shows how the true positive rate (sensitivity) and false positive rate (1-specificity) fluctuate with various thresholds. The performance of a random classifier (AUC = 0.5) is represented by the dashed diagonal line, while the performance of the proposed model is represented by the blue curve. The high AUC value of 0.96 amply illustrates the excellent discriminative power of the proposed method. The closer the AUC number is to 1.0, the more successfully it can differentiate between the various classes. A ROC curve that moves faster toward the upper left corner indicates a greater true positive rate and a lower false positive rate. Overall, the results of the ROC analysis demonstrate the robustness and efficacy of the proposed classifier in precisely identifying positive events with the fewest number of classification errors.

**Fig 11:**
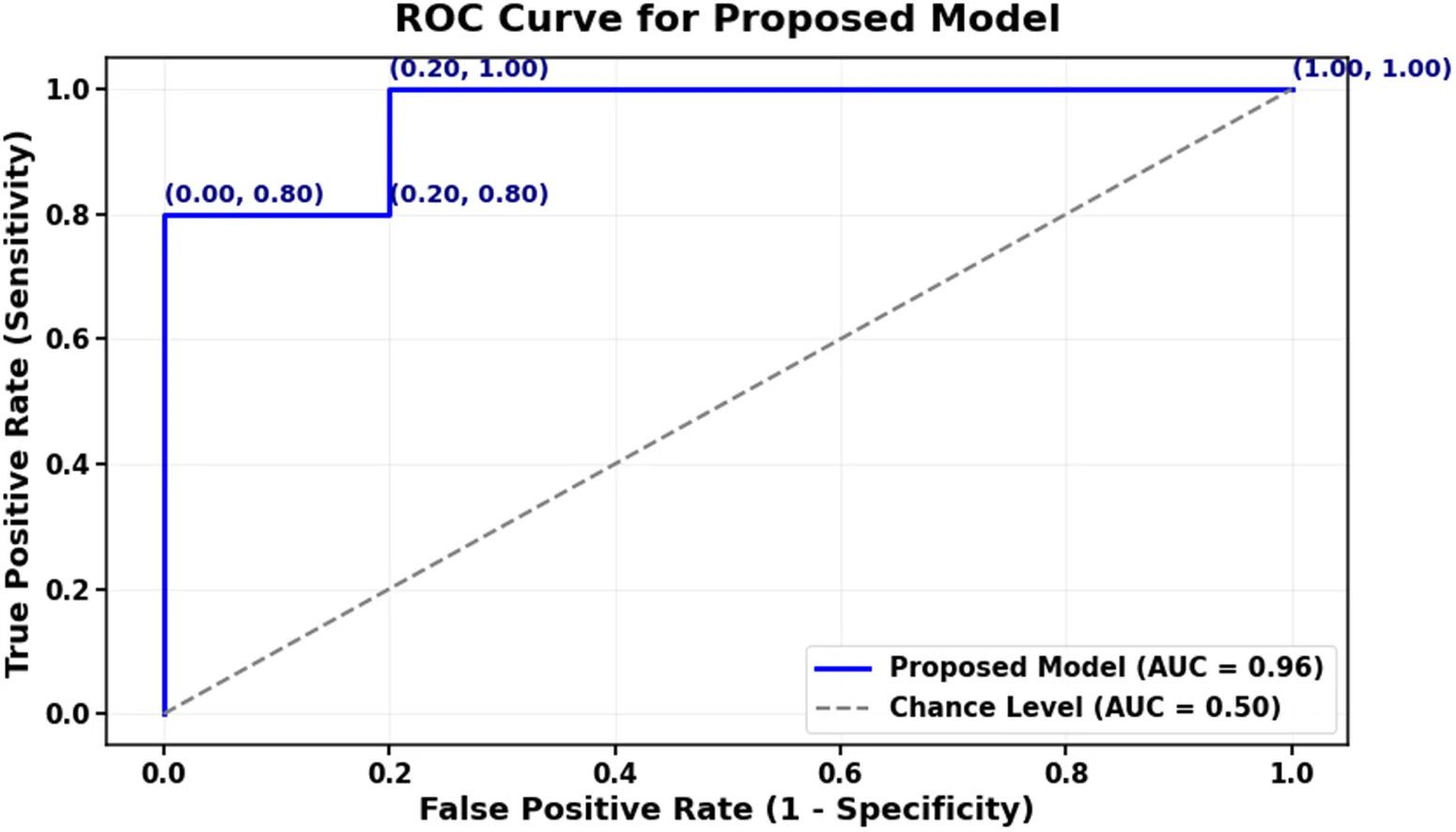
ROC for ReViT-RF model.

### Result metrics

The results of the classification of the proposed approach presented in Table 6 reflect that the approach has good classification performance. The model is able to correctly classify a large proportion of samples with an accuracy rate of 97.62%. The model precision is 96.91%, which means that it misclassifies very few positive cases. Moreover, the model can be used to detect the actual positive cases and fewest false negatives, with high recall rate of 96.13%. Also, the F1 score of the model (96.72%) shows that with an unbalanced data set, it still performs well without affecting the recall and precision. The high AUC–ROC value of 96.40% further confirms the model’s ability to classify different classes effectively, with its outstanding discriminative power. In conclusion, the results validate the proposed method and its applicability for other fields that require high accuracy in classification such as medical diagnosis and decision-making systems.

**Table 6:** Metrics of ReViT-RF model.

Metric	Value (%)
F1-Score	96.72
Recall	96.13
Accuracy	97.62
Precision	96.91
AUC-ROC	96.40

The reported performance metrics are patient-level evaluations seen through the slice-to-study aggregation with confidence weighted majority voting. The proposed ReViT-RF model showed to achieve a stable and reliable performance in patient-level TNM staging, with narrow patient-level confidence intervals, demonstrating good generalisation to independent patient studies.

### Metrics comparison with traditional model

Table 7 contrasts the effectiveness of the proposed ReViT-RF method with CNN-VIT, a hybrid deep learning model. Accuracy, precision, recall, and F1 score are evaluation metrics. When comparing the proposed ResNet-50 + ViT model to the existing hybrid models, the accuracy result is 97.62%, precision is 96.91%, recall is 96.13%, and F1score is 96.72%. The results show that the hybrid model can successfully lower the number of false positives and false negatives, improving the model’s capacity to discriminate between real and fake circumstances. Additionally, the model’s recall and precision are balanced. The DenseNet121 + ViT and ResNet101 + ViT models also perform similarly, with matching accuracy ratings of 96.55% and 96.45%. Furthermore, DenseNet121 + ViT demonstrates its effectiveness in feature representation and classification with an impressive F1 score of 94.94%.

**Table 7:** Comparison of metrics with various traditional model in combination with ViT.

Hybrid Model	Accuracy (%)	Precision (%)	Recall (%)	F1 Score (%)
SqueezeNet + ViT	93.20	91.50	90.80	86.75
EfficientNet + ViT	95.60	93.80	92.10	89.15
ResNet18 + ViT	95.10	94.25	93.40	90.02
ResNet101 + ViT	96.45	95.00	94.20	92.15
DenseNet121 + ViT	96.55	95.30	94.70	94.94
Proposed ResNet50 + ViT	97.62	96.91	96.13	96.72

With an F1 score of 86.75% and a precision of 93.20%, the SqueezeNet + ViT model performs poorly overall. Compared to more profound, and more expressive, convolutional neural network architectures, SqueezeNet has a limited ability to extract features, which may hinder its ability to capture complex and discriminative representations. The proposed ResNet-50 + Vision Transformer framework, however, has achieved better image classification results and learned robust and discriminative feature representations, outperforming the current hybrid architectures.

Fig 12 is used to compare the ViT models’ performances to those of other CNN-based hybrid deep learning models. The models’ performance is assessed using four key metrics: accuracy, precision, recall, and F1-score. With 97.62% accuracy, the suggested ResNet-50 + ViT model outperforms all evaluation measures, demonstrating its superior classification capacity. With a precision of 96.91% and an accuracy of 96.13%, the model accurately classifies the majority of genuine positive cases while having a low number of false positive cases. Additionally, the balanced precision and recall performance is shown by the F1 score of 96.72%. While ResNet101 + ViT and DenseNet121 + ViT are competitive hybrid models, they fall short of the suggested model. The outcomes demonstrate that the ResNet-50 + ViT model can also attain superior accuracy, robustness, and dependability, making it more appropriate for real-world classification applications.

**Fig 12:**
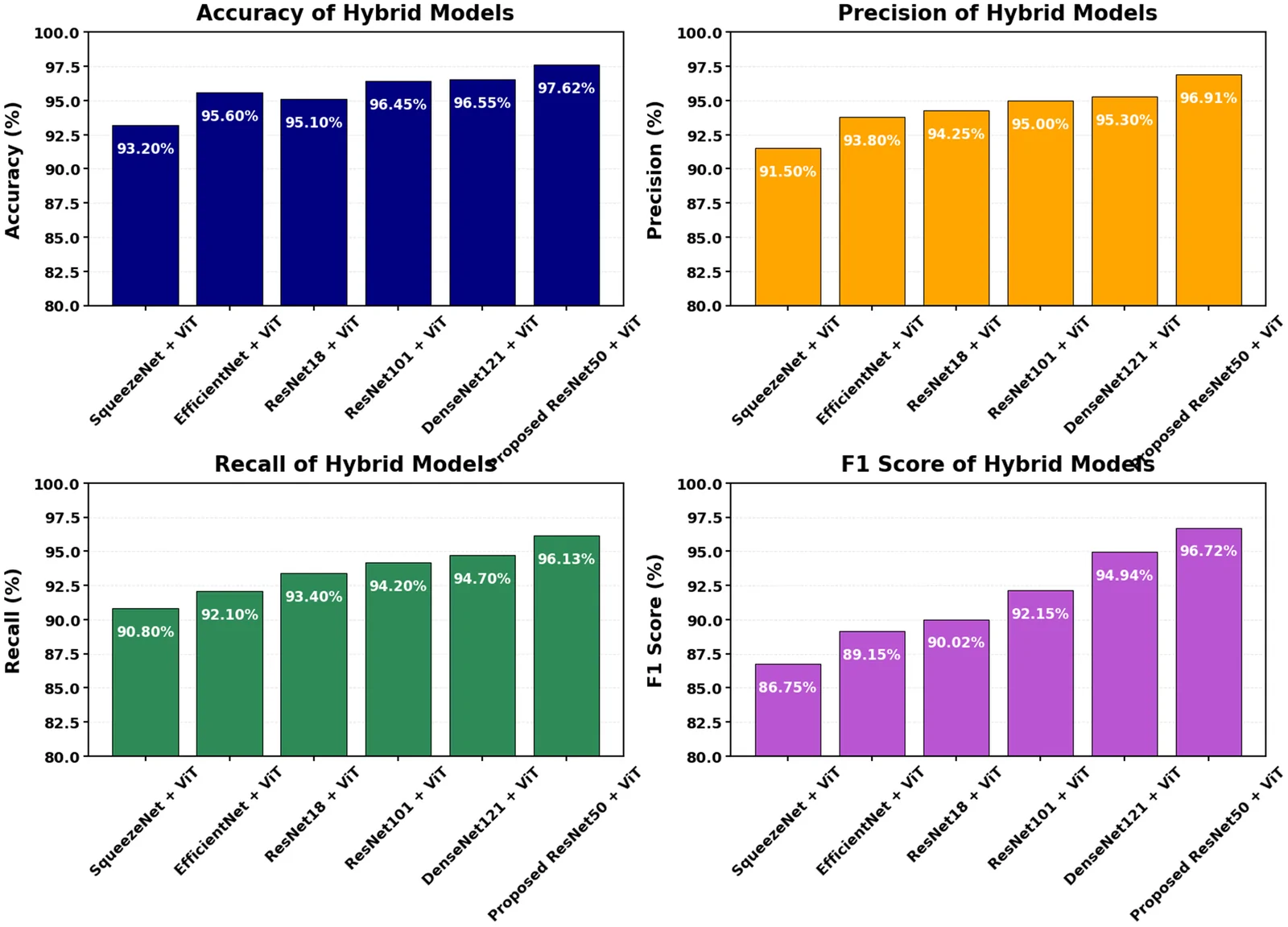
Evaluation of ReViT-RF model for lung tumor.

### Confusion matrix for TNM staging

The performance of the proposed ReViT-RF framework for binary classification of lung tumour cases (benign vs. malignant), is shown in the confusion matrix in Fig 13. With 205 benign and 210 malignant samples correctly categorised and a few misclassifications (three false positives and two false negatives), the matrix shows that the model obtained a high accuracy rate. The suggested hybrid ResNet-50–Vision Transformer design offers a good discriminative ability for discriminating tumour kinds, as seen by the high diagonal values. The model’s effectiveness in lowering the number of cancer cases that are overlooked is highlighted by the low false-negative rate, which is important for lung cancer early detection. In summary, the results indicate that a combination of local morphology, global context representations and radiomic data allow for the reliable and accurate classification of lung tumours through computed tomography (CT) imaging.

**Fig 13:**
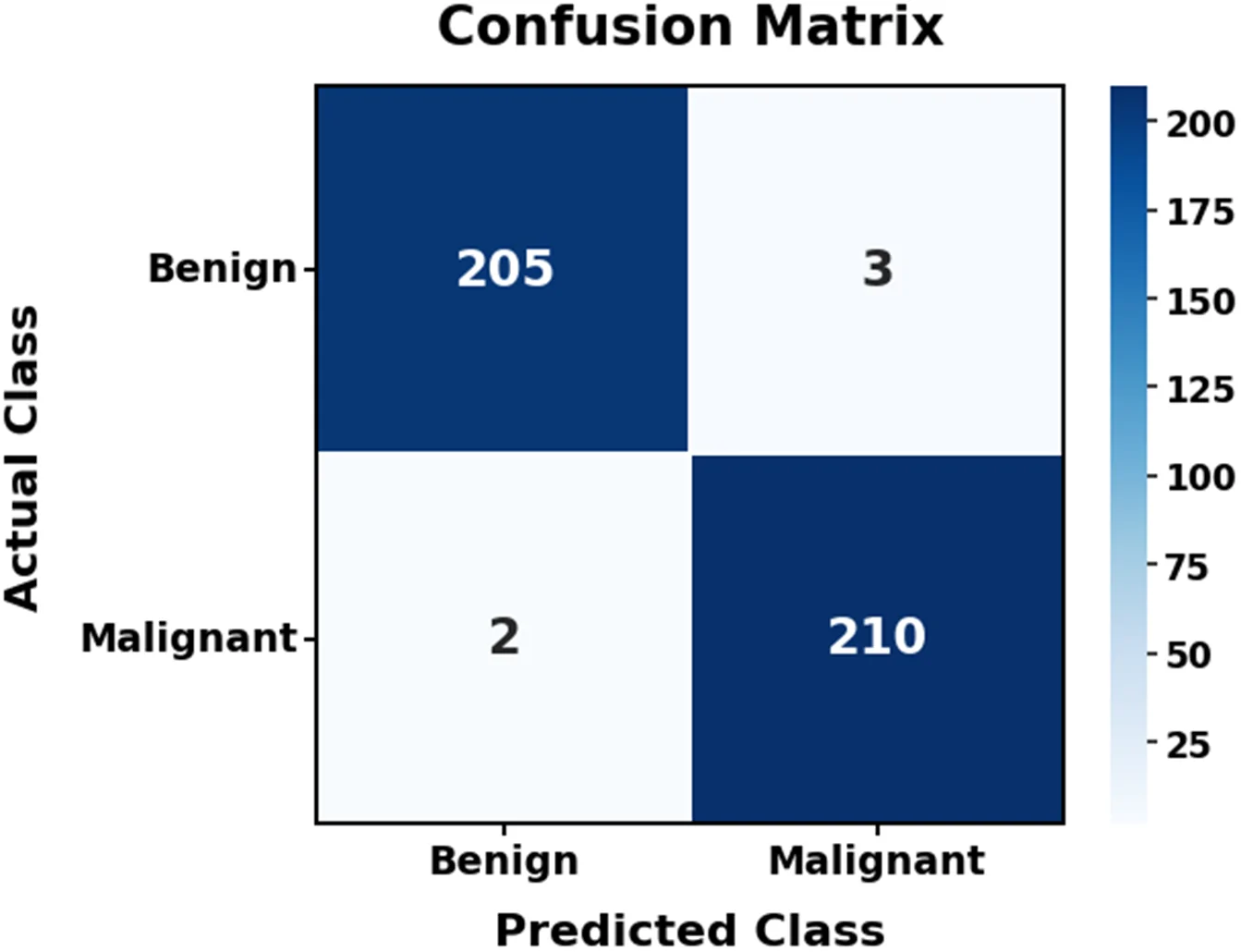
The proposed ReViT-RF model for benign–malignant lung tumor classification.

The TNM stage classification confusion matrix for the training set is shown on the left in Fig 14, and the confusion matrix for the test set is shown on the right. With the majority of the predictions grouped around the diagonal, the two datasets exhibit nearly flawless classification results. High classification accuracy is demonstrated by the trained model’s confusion matrix (left), which indicates that most samples are correctly identified with few misclassifications. The accuracy of classification for Stage 1A1 and Stage 4A/B is 99.0% and 97.0% respectively. The rest of the stages also achieve above 92.0% accuracy, showing the efficacy of the model in correctly categorising TNM stages.

**Fig 14:**
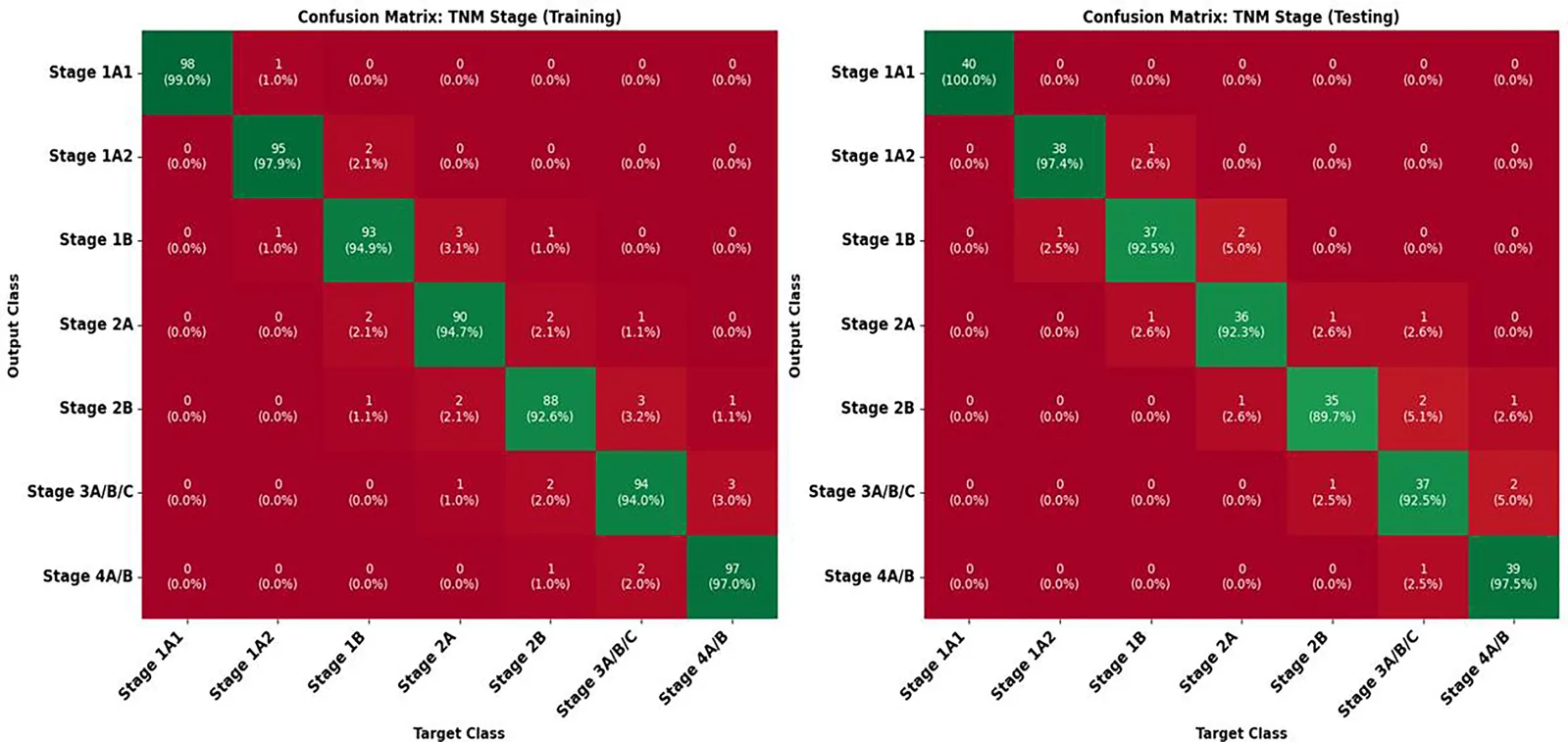
Confusion matrix of the TNM stage for training and testing.

This is also evident in the performance on the testing matrix (right) with a classification accuracy of 100% for Stage 1A1 and a classification accuracy greater than 89% for several other stages (including Stages 2B and 4A/B). There were some minor misclassifications (fewer than 10%) such as misclassifications between adjacent clinical stages (e.g., a few Stage 1B cases were misclassified as Stage 2A). But those errors were rare and readily understood. The confusion matrices show that the model performs well with minimal inter-class confusion and good accuracy across the different classes, especially between classes that are included in both the training and testing sets and have similar clinical presentations. This implies that the proposed model has a strong capacity for generalisation.

Fig 15 shows the computational efficiency evaluation of different hybrid CNN–ViT models regarding inference time, GPU memory usage and FLOPs. The proposed model ReViT-RF was evaluated with the smallest inference time of 20.1 ms/image and only 6.0 GB of the GPU memory with competitive computation complexity of 24.1 GFLOPs. Both DenseNet121 + ViT and ResNet101 + ViT, in comparison, needed more computational resources: 30.2 GFLOPs and 28.4 GFLOPs, respectively. The findings show that the recommended ReViT-RF model is a successful technique with a high classification accuracy for lung tumour investigation.

**Fig 15:**
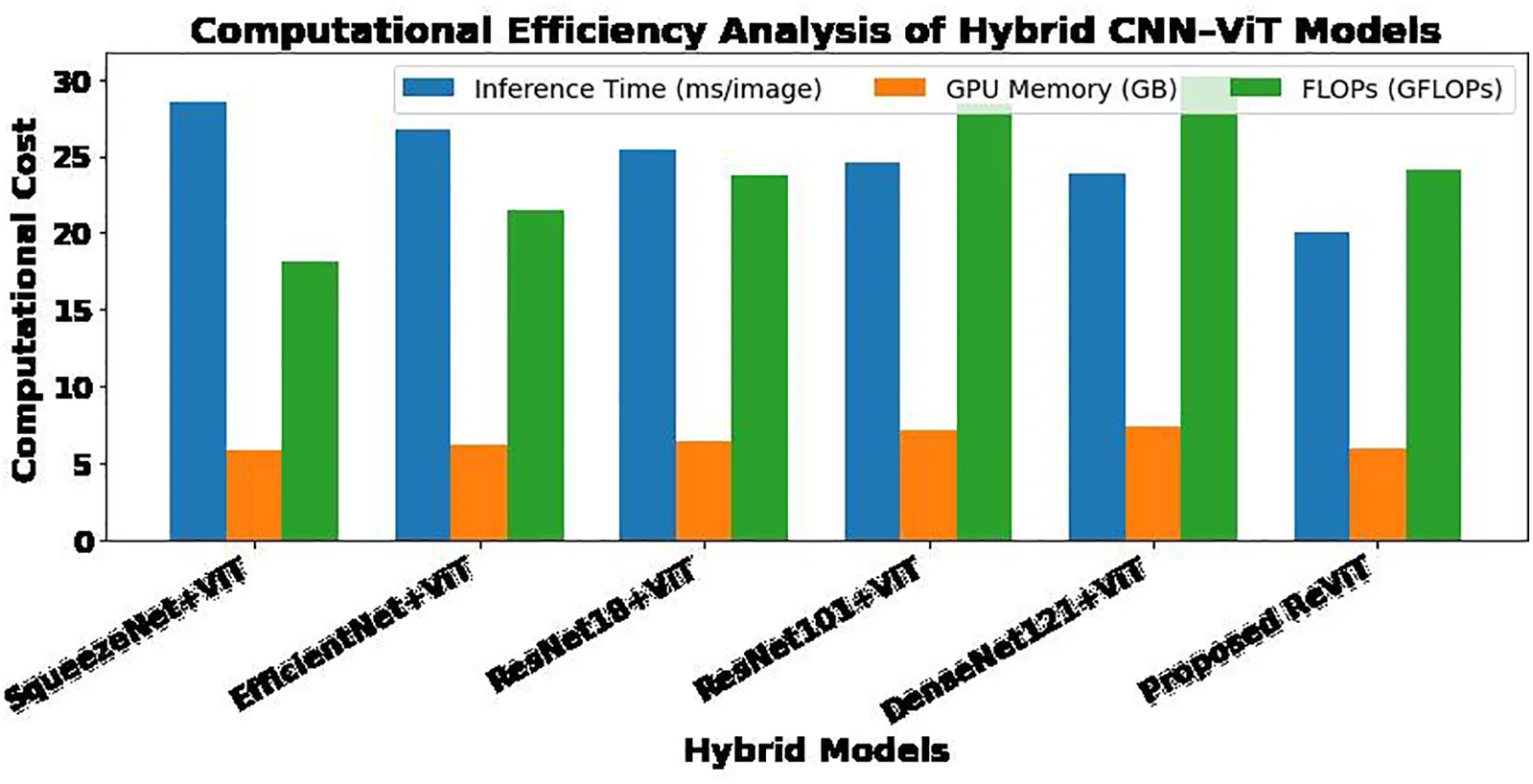
Computational efficiency analysis of hybrid CNN–ViT models.

### Ablation study

To determine whether the ResNet-50, Vision Transformer, and radiomic feature branches contain complementing information, an ablation research was conducted. The ResNet-50 standalone model worked well for detecting the local morphological characteristics but was not suitable for the treatment of heterogeneous appearances of tumors and complex spatial contexts. The standalone Vision Transformer was effective in capturing global dependencies, but did not show as much sensitivity to small tumor borders and texture. While the radiomic-only model could give interpretable quantitative descriptors, its discriminative power was restricted and handcrafted features were not able to fully capture the appearance variations in CT images that are complex.

By incorporating local structural information with global contextual dependencies, the combination of ResNet-50 and the Vision Transformer improved the overall performance. The classification and prediction of TNM components were further improved by the incorporation of radiomic features such that shape, intensity and texture descriptors from the GLCM offered explicit information regarding tumour size, irregularity, homogeneity, contrast, and heterogeneity. The intermediate fusion fusion strategy was identified as performing best when compared to early and late fusion strategies. While early fusion fused together heterogeneous features before adequate representation learning, late fusion fused only the final prediction scores without being able to well capture cross-modal interactions. Conversely, with intermediate fusion, each branch was trained to learn particular representations and then optimized for combined representation of their complementary parts.

The standalone ResNet-50 model performed well with an accuracy of 93.84% and the ViT model slightly outdid it with an accuracy of 94.61%, thanks to its better global context modeling ability, as presented in Table 8. Further integration of ResNet-50 with ViT further imbued the classification accuracy of 96.43%, where it was found that long-range context information and local morphological features are very complementary. The ReViT-RF framework with fusion of radiomic features has the highest overall performance with F1 score of 0.9711 and accuracy of 0.9762. The results support the proposed hybrid architecture for lung tumour classification and component-wise TNM prediction, where each architectural component is found to contribute significantly in improving the classification performance.

**Table 8:** Ablation Study of the Proposed ReViT-RF Framework.

Model Variant	ResNet-50	ViT	Radiomics	Accuracy (%)	Precision (%)	Recall (%)	F1-Score (%)
ResNet-50 Only	✓	✗	✗	93.84	93.21	92.96	93.08
ViT Only	✗	✓	✗	94.61	94.08	93.72	93.90
ResNet-50 + ViT	✓	✓	✗	96.43	96.02	95.76	95.89
ResNet-50 + Radiomics	✓	✗	✓	95.72	95.31	95.08	95.19
ViT + Radiomics	✗	✓	✓	96.18	95.82	95.37	95.59
Proposed ReViT-RF	✓	✓	✓	97.62	97.13	97.08	97.11

In Table 8 and Fig 16 it is shown that the contribution of each ResNet-50, ViT, and radiomic features is shown in the proposed ReViT-RF framework. The standalone ResNet-50 and ViT models achieved an accuracy of 93.84% and 94.61%, respectively, and their combination boosted the accuracy to 96.43%. The addition of radiomic information added complementary information about texture, shape and spatial characteristics, further improving the classification performance. The highest accuracy for the complete ReViT-RF model was 97.62% with an F1-score of 97.11%, showing its effectiveness and robustness in classification. The effectiveness in merging local CNN derived features, global transformer-based representations, and handcrafted radiomic descriptors is the reason for this improvement.

**Fig 16:**
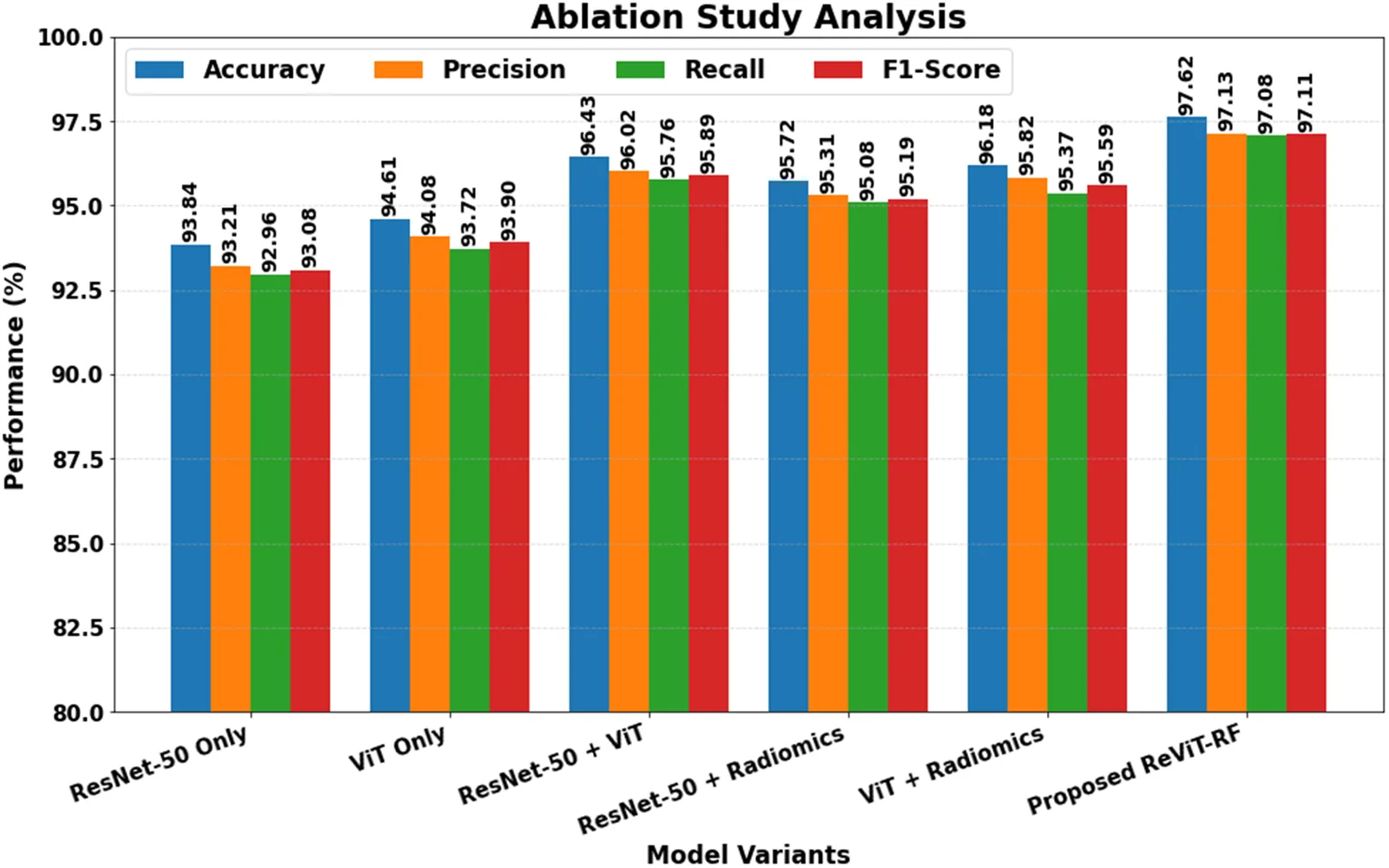
Ablation study of the proposed ReViT-RF framework.

## Discussion

All four metrics—accuracy, precision, recall, and robustness—showed notable improvements when the suggested ReViT-RF framework was compared to the other lung cancer classification techniques now in use. ResNet-50 and Vision Transformer work together to enable the model to efficiently extract both global contextual information and local spatial features from the CT images. Furthermore, the clinical interpretability and sensitivity for stage-level discriminating are improved by combining GLCM-based radiomic characteristics with explainability methods as Grad-CAM. The proposed framework is able to predict patient-level TNM stage without any patient-level leakage of slice information between training and testing partitions as well as enhancing the generalization capability and reliability for clinical applications in real world. This is a significant weakness found in some previous studies using slices.

Compare recently proposed deep learning based and hybrid lung cancer classification techniques in Table 9. The Modified U-Net + SVM, PCA-SMOTE-CNN, Res-U-Net and Attention-Based Capsule Networks obtained good classification results with accuracy ranging from 93.40% to 96.45%. With an accuracy of 97.62% for the LIDC-IDRI data set, the suggested ReViT-RF model outperformed the other models already in use, including ResNet50, ResNeXt, DenseNet, and VG-GAN. The overall increased performance demonstrates that accurate lung tumour classification and TNM staging may be achieved by combining CNN-based local feature extraction with transformer-based global contextual learning.

**Table 9:** State-of-the-art comparison with lung cancer classification methods.

Author & Reference	Methodology	Dataset	Accuracy (%)
Dunn et al. [12]	Radiomic Analysis + Deep Learning	Lung-PET-CT-Dx	94.20
Naseer et al. [13]	Modified U-Net + SVM	LIDC-IDRI	95.10
Mohamed and Ezugwu [14]	PCA-SMOTE-CNN	Multi-Omics Dataset	95.80
Wahengbam and Sriram [15]	CNN/ SAE/ RNN	CT Lung Dataset	93.40
Malligeswari and Kavya [16]	Res-U-Net + SVM	PET/CT Dataset	94.85
Kasinathan and Jayakumar [18]	Cloud-LTDSC + M-CNN	LIDC-IDRI	96.12
Paramasivam et al. [19]	Attention-Based Capsule Network	LIDC-IDRI	96.45
Proposed ReViT-RF Model	ResNet50 + Vision Transformer	LIDC-IDRI	97.62

The proposed framework is different from most of the previous slice-based studies because it integrates the prediction results of all CT slices for the patient level TNM diagnosis without influencing each other in the training and testing partitions, which means no information leakage occurs at the slice level.

The proposed framework presents a methodology limitation, as clinical TNM evaluation is based on a volumetric and multimodal evaluation. The current staging system is estimated from imaging; it is not a definitive pathological staging. Because of the lack of complete 3D volumetric modelling, clinical information (along the entire length of the tumour) and histopathological validation, staging may be affected, especially in lymph node involvement and distant metastasis. To ensure reliability and minimise slice-level data leakage, confidence-weighted slice aggregation and a strict patient-level data separation strategy was used. The proposed system was designed to assist the assessment process by imaging, and is not meant to replace the current TNM staging process. Future work will be directed towards incorporating full 3D CT volumes, PET/CT imaging and clinical metadata into the automated staging process to more recover the robustness, accuracy, and clinical effectiveness of automated staging.

The experiments show that the proposed ReViT-RF model combining the architecture of Vision Transformer and Convolutional Neural Network (ResNet-50) can not only provide a better performance in feature extraction but also provide a better generalization performance. The complementary nature of the convolutional and transformer-based representations is important, as they successfully capture local spatial features and global contextual data, respectively, resulting in outstanding classification performance. In this regard, the ReViT-RF model offers a very efficient and accurate classification solution that has high potential for use in real-world applications.

### Limitations

Accurate N- and M-stage prediction from slice-based CT analysis remains challenging because lymph nodes and distant metastatic lesions may not be fully visible in individual 2D thoracic CT slices. Therefore, the proposed framework provides imaging-based proxy estimations rather than definitive clinical TNM staging confirmation.

## Conclusion

In this paper, authors suggest a hybrid ReViT-RF framework that combines ResNet-50, Vision Transformer, radiomic features extracted from the GLCM, and explainability features derived from Grad-CAM to classify lung tumor and predict the imaging components of TNM classification using the LIDC-IDRI dataset. The suggested framework effectively combines global contextual learning with local spatial feature extraction to achieve stage-sensitive discrimination and benign–malignant classification. With an accuracy of 97.62%, precision of 96.91%, recall of 96.13%, F1-score of 96.72%, and AUC-ROC of 96.40%, experimental results demonstrate the superiority and generalisation capabilities of the suggested architecture. Additionally, each fold’s accuracy from the 10-fold cross validation ranges from 96.54% to 97.62%, demonstrating accuracy consistency throughout the 10 folds. When combined with explainable artificial intelligence through the activation of Grad-CAM, handcrafted GLCM radiomic features further promote the transparency and interpretability of the model, revealing the areas of tumor involvement that are important for the prediction results. The patient-level aggregation strategy, together with pairwise error analysis and slice-level data leakage prevention, further enhances the evaluation reliability and lessens the possibility of overfitting. One drawback of this study is the fact that TNM prediction is done solely on the basis of 2D slice analysis and proxy annotations of the images, instead of the complete pathological TNM ground truth. The developed framework should, therefore, be seen as a complementary system that supports the clinical diagnosis, rather than a substitute for the conventional clinical staging methods, since the complete volumetric evaluation of the tumor, evaluation of the lymph nodes, confirmation of metastasis, and integration of multimodal clinical data are required for accurate staging. Longitudinal follow-up clinical data and 3D volumetric CT analysis will be integrated to further improve staging accuracy and clinical applicability of the proposed approach in future work, along with incorporation of PET/CT imaging and histopathological validation. To conclude, the proposed ReViT-RF framework holds great promise for enhancing lung cancer detection, patient risk stratification, and clinical decision support by combining Deep learning and Explainable AI methods.
